# *n*-Propyl 6-amino-2,6-dideoxy-2,2-difluoro-β-d-glucopyranoside is a good inhibitor for the β-galactosidase from *E. coli*

**DOI:** 10.1007/s00044-021-02715-8

**Published:** 2021-03-05

**Authors:** Immo Serbian, Erik Prell, Claudia Fischer, Hans-Peter Deigner, René Csuk

**Affiliations:** 1 Martin-Luther-University Halle-Wittenberg, Organic Chemistry, Kurt-Mothes_Str. 2, D-06120 Halle (Saale), Germany; 2 Department of Radiation Medicine, Section of Nuclear Medicine, University Hospital Halle (Saale), Ernst-Grube Str. 40, D-06120 Halle (Saale), Germany; 3 Medical and Life Sciences Faculty, Furtwangen University, Jakob-Kienzle Str. 17, D-78054 Villingen-Schwenningen, Germany

**Keywords:** Fluorinated monosaccharides, Glucosidase, Galactosidase, Inhibitor

## Abstract

A convenient route has been developed for the synthesis of novel 6-amino-2,2-(or 3,3-difluoro)-2-(or 3),6-dideoxy-hexopyranoses. Biological screening showed these compounds as good inhibitors for several glycosidases. Especially *n*-propyl 6-amino-2,6-dideoxy-2,2-difluoro-β-d-glucopyranoside (**8**) was an excellent competitive inhibitor for the β-galactosidase from *E. coli* holding a *K*_*i*_ of 0.50 μM.

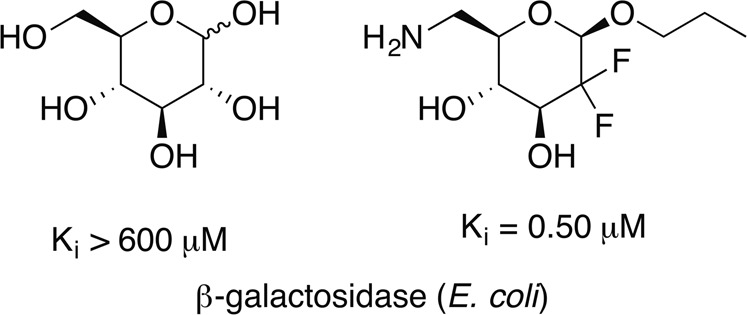

## Introduction

**Fig. 1:**
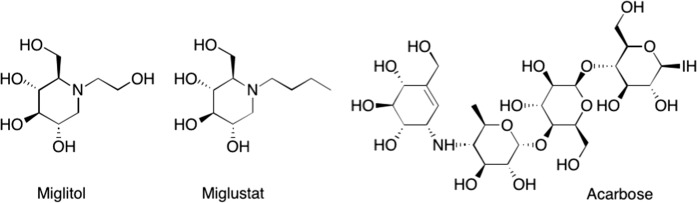
Structure of glucosidase inhibitors miglitol and miglustat and acarbose

Glycosidases are involved in many important biological processes, and inhibitors of glycosidases [1–3] are discussed as therapeutics for diabetes mellitus, lysosomal storage diseases, and obesity [4–7]. Furthermore, nitrogen-containing [8] glycomimetics such as aza- or iminosugars are known to behave as pharmacological pharmacophores for lysosomal storage disorders [9]. Miglitol (Fig. 1) is an α-glucosidase inhibitor; this enzyme catalyzes the hydrolysis of oligo-, tri-, and disaccharides to glucose and other monosaccharides in the intestine. Thereby, an increased blood sugar level (as usually observed after meals) is reduced. Hence, miglitol is in principle suitable for the therapy of diabetes mellitus type 2 [10–13]. In Germany, it was in use until 2015 but is nowadays often replaced by acarbose, a pseudotetrasaccharide. An analog of miglitol, miglustat, is a drug used to treat type I Gaucher disease [14–19] and was the first treatment to be approved for treating progressive neurological complications in people with Niemann–Pick disease [20–28]. Recently, miglustat has been suggested for the therapy of COVID-19, too [29, 30].

An extended search in literature as of 2020, October, revealed there are some 30,600 structures containing the substructure of a 6-amino-6-deoxy-hexopyranose (or -pyranoside). Monofluorinated analogs, however, have scarcely been prepared (some glycosyl fluorides and a couple of 2-fluoro and 3-fluoro analogs; the latter—by and large—being part of modified kanamycin derivatives) [31–37]—but to the best of our knowledge, there are no 2,2- or 3,3-difluoro-6-amino-2 (or 3), 6-dideoxy-hexopyranoses (respectively -hexopyranosides). Some molecules containing the structural element of a 6-amino-hexose have been proposed in-silico as possible lead structures for the therapy of COVID-19 diseases [38–40]. Therefore, we set out for the first synthesis of these targets and to test their ability to act as glycosidase inhibitors. It is reserved for later studies to have a look at their properties regarding lysosomal storage diseases or antiviral activity.

## Results and discussion

**Scheme 1:**
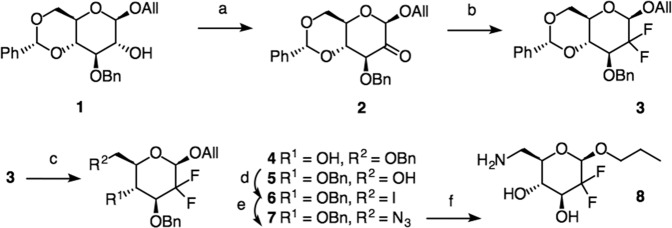
Synthesis of 6-amino-2,6-dideoxy-2,2-difluoro-hexopyranoside **8**: (a) DMSO, TFAA, DCM, −78 → 25 °C, 12 h, 89%; (b) DAST, DCM, 25 °C, 5 days, 76%; (c) LiAlH_4_/AlCl_3_, Δ, 48 h; 4 (15%), 5 (50%); (d) PPh_3_, imidazole, I_2_, 90 °C, 2 h, 52%; (e) LiN_3_, DMF, 25 °C, 4 days, 89%; (f) Pd/C (10%), H_2_ (30 °C, 2.43 atm), 48 h, 88%

Swern oxidation [41] (Scheme 1) of well-known allyl glycoside **1** [42] gave 89% of the ulose **2** whose difluorination with DAST [43] in dichloromethane for 5 days gave 76% of difluorinated 3. Compound 3 was treated with LiAlH_4_/AlCl_3_ [44] to open the benzylidene acetal, and a mixture of **4** and **5** was obtained. Product **5** was iodinated using in situ prepared triiodoimidazole [45–47] resulting in the formation of 52% of 6-iodo **6**. Nucleophilic displacement with lithium azide [45] for 4 days provided 89% of 6-azido **7** whose hydrogenation gave 88% of *n*-propyl-6-amino derivative **8**.

**Scheme 2:**
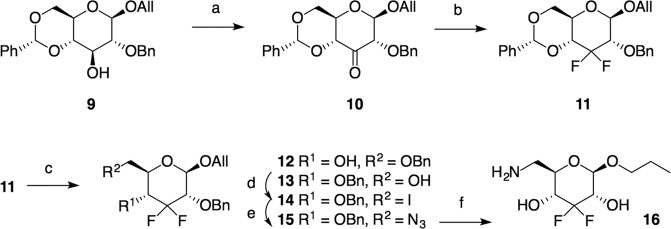
Synthesis of 6-amino-3,6-dideoxy-3,3-difluoro-hexopyranoside **16**: (a) DMSO, TFAA, DCM, −78 → 25 °C, 12 h, 75%; (b) DAST, DCM, 25 °C, 5 days, 55%; (c) LiAlH_4_/AlCl_3_, Δ, 48 h; 12 (43%), 13 (12%); (d) PPh_3_, imidazole, I_2_, 90 °C, 2 h, 78%; (e) LiN_3_, DMF, 25 °C, 4 days, 77%; (f) Pd/C (10%), H_2_ (30 °C, 2.43 atm), 48 h, 93%

For the synthesis of the 3, 3-difluoro compounds, **9** [48] (Scheme 2) served as a starting material whose Swern oxidation [41] (→ **10**) followed by difluorination [43] with DAST gave 82% of difluoro **11**.

Partial deprotection [44] of **11** gave a mixture of **12** and **13**. Main component **12** was iodinated as described above to afford **14** whose subsequent nucleophilic displacement reaction (→ **15**) and hydrogenation gave glycoside **16**.

Low or absent cytotoxicity is a mandatory requirement for subsequent therapeutic use of the compounds as glycosidase inhibitors. Therefore, their cytotoxicity was determined. Compounds **8** and **16** were tested for antitumor activity in a panel of 15 human cancer cell lines (518A2, A-431, A-253, FaDu, A-549, A-2780, DLD-1, HCT-8, HCT-116, HAT-29, SW480, 8505-C, SW1736, MCF-7, and Lipo) and for cytotoxicity on nonmalignant NIH 3T3 mouse fibroblasts (using a sulforhodamine B (SRB) assay) [49] but no significant cytotoxicity was observed (IC_50_ > 50 μM).

An in vitro evaluation of these compounds to act as an enzyme inhibitor using a panel of commercially available glycosidases was performed (Table 1) using p-nitrophenolate [50] assays.

**Table 1:** Glycosidase inhibition (*K*_*i*_ in μM from *p*-nitrophenolate assays (mean of experiments performed in triplicate with three technical triplicates, each; n.a. no activity); [50])

Enzyme	From	Miglitol	8	16
α-glucosidase	*B. stearothermophilus*	0.15 ± 0.03	2.21 ± 0.11	n.a.
α-glucosidase	Baker’s yeast	9.92 ± 0.14	1.83 ± 0.16	4.13 ± 0.26
β-glucosidase	Almonds	0.21 ± 0.05	1.76 ± 0.13	n.a.
α-galactosidase	Green coffee beans	13.90 ± 0.15	3.81 ± 0.57	4.30 ± 0.24
β-galactosidase	*E. coli*	0.16 ± 0.06	0.50 ± 0.09	2.16 ± 0.93

Compounds **8** and **16** were competitive inhibitors for all enzymes. Thereby, compound **8** was a nonselective inhibitor, and its highest activity was found for the ß-galactosidase from *E. coli*. Glycoside **16** was a weaker inhibitor than **8**.

For a better interpretation of these results additional molecular modeling calculations were carried out. Thereby, the protein crystal structures were selected on their genetic similarity to the enzymes employed in the in vitro experiments. An additional focus was set to a good resolution of the enzyme structure. This was easily achieved for several of the enzymes, while for the β-glucosidase from almonds and the α-galactosidase (from green coffee beans) an additional search for an enzyme from a different organism was called for. This was performed utilizing UniProt.org eventually finding enzymes of high similarity. Thus, miglitol and compounds **8** and **16** were evaluated in molecular docking studies against the enzymes α-glucosidase (*Geobacillus* sp.; PDB: 2ZE0, Baker’s yeast; PDB: 3AXI), β-glucosidase (from rice due to its similarity with the β-glucosidase from almonds; PDB: 1UAS), α-galactosidase (from white clover due to its similarity to the enzyme from green coffee beans; PDB: 1CBG), and β-galactosidase (*E. coli*; PDB: 1JYW). From the docking studies the binding affinity was estimated.

A rough blind docking of 50 individual runs showed that for compounds **8** and **16** their main binding site at the enzymes is similar to the binding site of miglitol in each case. Therefore, the search space was limited to the active site of the respective enzyme. As a result, five top poses were obtained being very closely located to each other holding H-bond interactions with closely related amino acids.

Thus, the 2,2- and 3,3-difluorinated miglitol analogs are efficient competitive inhibitors of the α- and β-glucosidase as well as of the galactosidases. Miglitol, however, showed lowered inhibition for yeast’s and green coffee bean’s α-glucosidase and galactosidase, while for compound **8** low *K*_*i*_ values for all tested enzymes were established. This is most likely due to favored interaction of the difluorinated compounds in the active sites leading eventually to better binding affinities for compounds **8** and **16** with yeast α-glucosidase and green coffee beans α-galactosidase (cf. Tables 2 and 3). Table 3 shows the main interactions of the compounds with the amino acids of the respective enzyme.

**Table 2:** Results from the molecular docking; mean ΔG in kcal/mol of the top 5 docking poses (standard deviation, median) for miglitol (standard) and compounds **8** and **16**

Enzyme	Miglitol	8	16
*Geobacillus* sp. (α-glucosidase)	−3.81 (0.11, −3.82)	−4.22 (0.05, −4.22)	−4.66 (0.31, −4.64)
Baker’s yeast (α-glucosidase)	−3.62 (0.48, −3.30)	−4.21 (0.02, −4.20)	−4.50 (0.10, −4.49)
Rice (β-glucosidase)	−5.12 (0.27, −5.01)	−5.53 (0.11, −5.52)	−5.32 (0.22, −5.39)
White clover (α-galactosidase)	−4.81 (0.01, −4.82)	−5.34 (0.10, −5.32)	−5.56 (0.07, −5.57)
*E. coli* (β-galactosidase)	−5.19 (0.17, −5.10)	−5.61 (0.15, −5.71)	−5.68 (0.14, −5.62)

**Table 3:** Main interactions of the compounds with the amino acids of the respective enzyme (ascending order, non-H-bonding interactions are in italic; equal residues (bold) of binding of miglitol and **8** or **16**, respectively)

	*Geobacillus* sp. (α-glucosidase)	Baker’s yeast (α-glucosidase)	Rice (β-glucosidase)	White clover (α-galactosidase)	*E. Coli* (β-galactosidase)
**8**	Gly62, Gln167, *Phe163*	Gln67, Pro66, Glu405, Glu408, **Trp468**	Asn190, *Phe277*, Ile344	**Trp16**, Asn17, Trp108, Arg181, Ile186,	**Asn102**, Gln537, His540, Trp568, Phe601
**16**	Gly259, *Phe282*, Arg411,	Pro66, Glu405, Lys406, Tyr407, Arg413, **Trp468**	Gln33, **Lys58**, Lys60, Asn182, Thr196, **His256**, Gln345, **Trp454**,	**Trp16**, Ile23, Cys21, **Trp164**, Ile186,	**Asn102**, **Gly489**, Val515, **Lys517**, Gln537, His540, Trp568,
Miglitol	Gly145, Arg197, Ser224, His325	Lys466, **Trp468**	**Lys58**, **His256**, Gly274, Trp369, **Trp454**	**Trp16**, His18, Lys104, **Trp164**, Asn222	**Asn102**, **Gly489**, **Lys517**, Gln537

The good binding affinities of compounds **8** and **16** as compared to miglitol allow no explanation for the low *K*_*i*_ value of miglitol for the α-glucosidase from *Geobacillus* sp. (almond’s β-glucosidase) and *E. coli*’s β-galactosidase. We assume that these findings are caused by different residence times of the compounds. As a consequence of this dynamic effect, the hydroxyl groups of miglitol may help to hold this compound in the periphery of the binding site thereby increasing the residence time of miglitol as compared to the residence time of compounds **8** and **16**. Comparing the docking results from Table 2, compound **8** seems to be a slightly better inhibitor than miglitol (−0.47 kcal/mol on average), and compound **16** seems to be also a slightly better inhibitor than compound **8** (−0.16 kcal/mol on average).

Table 3 summarizes the main binding residues thus allowing some insight into the different binding motifs of compounds **8**, **16** and miglitol. For the α-glucosidase from Baker’s yeast, Trp468 seems to be a common amino acid residue all evaluated compounds share H-bonding with, whereas other amino acid residues may vary. Furthermore compound **16** and miglitol share similar H-bonding motif; for example, docking of miglitol, **8**, and **16** with the β-glucosidase from rice showed miglitol and **16** to share Lys58, His256, and Trp454 as amino acid residues with H-bonding interactions. The results for the β-galactosidase from *E. coli* revealed that miglitol and compound **8** only share Asn102, whereas **16** formed H-bonds to Asn102, Gly489, and Lys517 as also observed for miglitol.

**Fig. 2:**
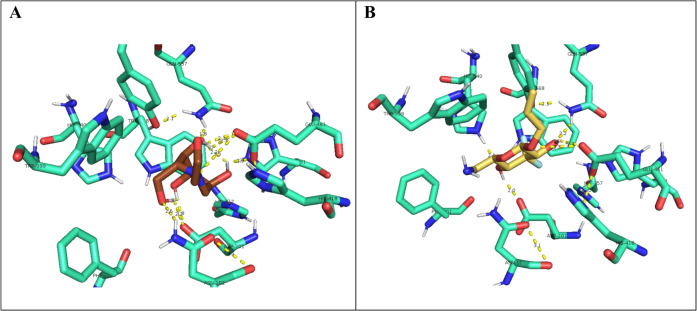
Interaction of miglitol (**A**) and of compound **16** (**B**) in the active site of β-galactosidase (*E. coli*) holding a *K*_*i*_ of 0.16 μM

Figure 2 depicts some of the results from the modeling, the interaction of miglitol and of compound **16** in the active site of the β-galactosidase from *E. coli*.

## Conclusion

*n*-Propyl 6-amino-2,6-dideoxy-2,2-difluoro-β-d-glucopyranoside (**8**) as well as its 3,3-difluor-analog (**16**) were easily accessible from allyl glycosides **1** and **9** by a sequence of oxidation, difluorination, selective deprotection, nucleophilic displacement, and catalytic hydrogenation. While no significant cytotoxicity was observed in the SRB assays (EC_50_ > 50 μM), screening of these compounds in *p*-nitrophenolate assays showed especially compound **8** as a good inhibitor for the β-galactosidase from *E. coli*. It also seems to be obvious for this novel class of compounds that the presence of a hydrophobic moiety in β-position has a stronger influence on the inhibitory activity with respect to β-galactosidase of *E. coli* than a corresponding hydrophobic moiety in γ-position.

## Experimental

Melting points are uncorrected (*Leica* hot stage microscope), optical rotations were obtained using a Perkin-Elmer 341 polarimeter (1 cm micro cell), NMR spectra were recorded using the Varian spectrometers Gemini 2000 or Unity 500 (*δ* given in ppm, *J* in Hz, internal Me_4_Si or internal CCl_3_F), IR spectra (film or KBr pellet) on a Perkin-Elmer FT-IR spectrometer Spectrum 1000, MS spectra were taken on a Intectra GmbH AMD 402 (electron impact, 70 eV) or on a Thermo Electron Finnigan LCQ (electrospray, voltage 4.5 kV, sheath gas nitrogen) instrument; for elemental analysis a Foss-Heraeus Vario EL instrument was used; TLC was performed on silica gel (Merck 5554, detection by UV absorption or by treatment with a solution of 10% sulfuric acid, ammonium molybdate, and cerium(IV) sulfate) followed by gentle heating. The solvents were dried according to usual procedures.

### Molecular docking experimental

The enzyme structure was accessed through http://rcsb.org (α-glucosidase (*Geobacillus* sp.; PDB: 2ZE0, Baker’s yeast; PDB: 3AXI), β-glucosidase (rice due to its similarity with almond β-glucosidase; PDB: 1UAS), α-galactosidase (white clover due to its similarity to green coffee beans; PDB: 1CBG), and β-galactosidase (*E. Coli*; PDB: 1JYW)). The structures were prepared with autodock tools 1.5.6 following usual procedures. The grid and docking files of the inhibitors and enzymes were also prepared with autodock tools 1.5.6. The grid spacing was set to 0.25 A. The genetic algorithm settings were 150 m populations size, 4 m evaluations, and 27k generations. Docking settings were 50 dockings of each individual inhibitor/enzyme pair with 4 m evaluations each. The corresponding file formats were prepared with open babel 2.4.1. Drawing and interactions were analyzed with PyMOL 2.0.6 from Schroedinger LLC.

### Glycosidase assays

The corresponding *p*-nitrophenyl glycoside and the inhibitors were dissolved in NaOAc buffer (0.05 m, pH = 6.0) reaching final concentrations of 1.0, 1.11, 1.25, 1.43, 1.67, 2.0, 2.5, 3.33, 5.0, and 10 mM. Further dilutions (if necessary) were made in the ratios 1:60, 1:50, 1:40, 1:30, 1:20, 1:10, and 1:5. The ice-cooled solution of the enzymes was contained between 4.0 and 4.5 U/mL. The kinetic assays were performed in 96-well microtiter plates. The following solutions were pipetted per well: *p*-nitrophenyl glycoside (100 μL), enzyme (50 μL), inhibitor (100 μL), and standard (*p*-nitrophenol, 5 μL). Immediately after addition, UV/Vis absorption was measured at 42 °C and *λ* = 415 nm for 20 min with a single measurement interval of 50 s.

### Cytotoxicity assay (SRB assay)

The cell lines were obtained from Department of oncology (Martin-Luther-University Halle-Wittenberg). Cultures were maintained as monolayers in RPMI 1640 medium with L-glutamine (Capricorn Scientific GmbH, Ebsdorfergrund, Germany) supplemented with 10% heat inactivated fetal bovine serum (Sigma-Aldrich Chemie GmbH, Steinheim, Germany) and penicillin/streptomycin (1%, Capricorn Scientific GmbH, Ebsdorfergrund, Germany) at 37 °C in a humidified atmosphere with 5% CO_2_.

The cytotoxicity of the compounds was evaluated using the SRB (Kiton-Red S, ABCR) micro culture colorimetric assay using confluent cells in 96-well plates with the seeding of the cells on day 0 applying appropriate cell densities to prevent confluence of the cells during the period of the experiment. On day 1, the cells were treated with six different concentrations (1, 3, 7, 12, 20, and 30 μM); thereby, the final concentration of DMSO was always < 0.5%, generally regarded as nontoxic to the cells. On day 4, the supernatant medium was discarded; the cells were fixed with 10% trichloroacetic acid. After another day at 4 °C, the cells were washed in a strip washer and dyed with the SRB solution (100 μL, 0.4% in 1% acetic acid) for about 20 min to be followed by washing of the plates (four times, 1% acetic acid) and air-drying overnight. Furthermore, tris base solution (200 μL, 10 mM) was added to each well and absorbance was measured at *λ* = 570 nm employing a reader (96-wells, Tecan Spectra, Crailsheim, Germany). The IC_50_ values were averaged from three independent experiments performed each in triplicate calculated from semi logarithmic dose response curves applying a nonlinear four-parameter Hills-slope equation (GraphPad Prism5; variables top and bottom were set to 100 and 0, respectively).

#### Allyl 3-O-benzyl-(R)-4,6-O-benzylidene-β-d-glucopyranoside (**1**) and allyl 2-O-benzyl-(R)-4,6-O-benzylidene-β-d-glucopyranoside (**9**)

To a solution of allyl (R) 4,6-O-benzylidene-β-d-glucopyranoside [42] (8.44 g, 27.27 mmol) and tetrabutylammonium hydrogen sulfate (1.63 g, 4.80 mmol) in dry DCM (315.0 mL) an aq. solution of sodium hydroxide (1.3 m, 31.6 mL) was added. At reflux temperature, benzyl bromide (4.91 mL, 41.06 mmol) was added dropwise and stirring was continued for 96 h. Usual aqueous workup followed by chromatography (silica gel, *n*-hexane/ethyl acetate, 5:15) gave **1** (2.71 g, 25%) and **11** (5.22 g, 48%).

Data for **1**: white solid; MP 139–140 °C (lit.: [51] 140–141 °C); [*α*]_*D*_ = −18.96° (*c* 0.42, CHCl_3_); [lit.: [51] [*α*]_*D*_ = +39.9° (*c* 1, CHCl_3_)]; *R*_*ƒ*_ (*n*-hexane/ethyl acetate, 5:3) = 0.53; analysis calcd for C_23_H_26_O_6_ (398.45): C 69.33, H 6.58; found: C 69.09, H 6.63.

Data for **9**: white solid; MP 124–125 °C (lit.: [48] 124 °C); [*α*]_*D*_ = −17.70° (*c* 0.93 g, CHCl_3_); *R*_*ƒ*_ (*n*-hexane/ethyl acetate, 5:3) = 0.63; analysis calcd for C_23_H_26_O_6_ (398.45): C 69.33, H 6.58; found: C 69.17, H 6.71.

#### Allyl 3-*O*-benzyl-(*R*)-4,6-*O*-benzylidene-β-d-arabino-hexopyranoside-2-ulose (**2**)

To a solution of dry DMSO (4.67 g, 59.80 mmol) in dry DCM (40.0 mL) at −78 °C, a solution of trifluoroacetic anhydride (8.92 g, 42.45 mmol) in dry DCM (10.0 mL) was slowly added dropwise, and the mixture was stirred at this temperature for 45 min followed by adding a solution of **1** (8.14 g, 20.43 mmol) in dry DCM (40.0 mL) while maintaining the temperature at −78 °C. The mixture was stirred for 2 h at −78 °C, then a solution of Et_3_N (9.0 mL, 64.57 mmol) in dry DCM (40.0 mL) was added dropwise. The mixture was stirred at −78 °C for another 30 min and at 25 °C for 12 h. Usual aqueous workup followed by chromatography (silica gel, *n*-hexane/ethyl acetate, 5:3) gave **2** (7.18 g, 89%) as a white solid; MP 130–132 °C; [*α*]_*D*_ = −47.95° (*c* 0.73, CHCl_3_); *R*_*ƒ*_ (*n*-hexane/ethyl acetate, 5:3) = 0.40 (ketone); *R*_*ƒ*_ (hexane/ethyl acetate, 5:3) = 0.19 (hydrate); analysis calcd for C_23_H_24_O_6_ (396.43): C 69.68, H 6.10; found: C 69.43, H 6.27.

#### Allyl 3-O-benzyl-(R)-4,6-O-benzylidene-2-deoxy-2,2-difluoro-β-d-arabino-hexopyranoside (**3**)

To a solution of **2** (300 mg, 0.72 mmol) in dry DCM (6.0 mL), DAST (417 μL 3.03 mmol) was added dropwise under argon, and the mixture was stirred at 25 °C for 5 days. Methanol (1.0 mL) was carefully added, and the solvents were removed under diminished pressure. The oily residue was dissolved in DCM (90 mL) and washed with water (50 mL). The aqueous phase was re-extracted with DCM (3 × 100 L); the organic layers were combined, and the solvent was evaporated under reduced pressure. The remaining residue was subjected to chromatography (silica gel, *n*-hexane/ethyl acetate, 85:15) to afford **3** (230 mg, 76%) as a white solid; MP 79–81 °C; [*α*]_*D*_ = −26.10° (*c* 0.71, CHCl_3_) *R*_*ƒ*_ (*n*-hexane/ethyl acetate, 5:3) = 0.75; analysis calcd for C_23_H_24_O_5_F_2_ (418.43): C 66.02, H 5.78; found: C 65.85, H 5.92.

#### Allyl 3,6-di-O-benzyl-2-deoxy-2,2-difluoro-β-d-arabino-hexopyranoside (**4**) and allyl 3,4-di-O-benzyl-2-deoxy-2,2-difluoro-β-d-arabino-hexopyranoside (**5**)

To an ice-cold solution of **3** (3.00 g, 7.17 mmol) in dry ether (30 mL) and THF (30 mL), lithium aluminum hydride (490 mg, 12.91 mmol) was added in several portions, and the suspension was stirred for 15 min at 25 °C. Then, the suspension was heated to reflux, and a solution of dry aluminum chloride (1.63 g, 12.19 mmol) in dry ether (30 mL) was added dropwise followed by stirring under reflux for another 48 h. The suspension was cooled to 25 °C, methanol (30 mL) was carefully added, and stirring was continued for another 30 min. After usual aqueous workup followed by chromatography (silica gel, *n*-hexane/ethyl acetate, 5:3) **4** (450 mg, 15%) and **5** (1.50 g, 50%) were obtained each as a colorless oil.

Data for **4**: [*α*]_*D*_ = −48.23° (*c* 0.44, CHCl_3_); *R*_*ƒ*_ (*n*-hexane/ethyl acetate, 5:3) = 0.51; analysis calcd for C_23_H_26_O_5_F_2_ (420.45): C 65.70, H 6.23; found: C 65.55, H 6.40.

Data for **5**: [*α*]_*D*_ = −18.27° (*c* 0.52, CHCl_3_); *R*_*ƒ*_ (*n*-hexane/ethyl acetate, 5:3) = 0.41; analysis calcd for C_23_H_26_O_5_F_2_ (420.45): C 65.70, H 6.23; found: C 65.47, H 6.39.

#### Allyl 3,4-di-O-benzyl-2,6-dideoxy-2,2-difluoro-6-iodo-β-d-arabino-hexopyranoside (**6**)

To a solution of **5** (1.50 g, 3.57 mmol) in toluene (30 mL) containing triphenylphosphane (2.06 g, 7.85 mmol) and imidazole (1.09 g, 16.06 mmol), iodine (1.81 g, 7.14 mmol) was added in several portions. After stirring at 90 °C for 2 h, the reaction mixture was decanted, and the remaining oil was washed with ether (3 × 100 mL). The combined organic layers were evaporated, and the remaining residue was subjected to chromatography (silica gel, *n*-hexane/ethyl acetate, 85:15) to afford **6** (900 mg, 52%) as a colorless oil; [*α*]_*D*_ = −25.32° (*c* 0.44, CHCl_3_); *R*_*ƒ*_ (*n*-hexane/ethyl acetate, 85:15) = 0.56; analysis calcd for C_23_H_25_O_4_F_2_I (530.34): C 52.09, H 4.75; found: C 51.84, H 4.92.

#### Allyl 6-azido-3,4-di-O-benzyl-2,6-dideoxy-2,2-difluoro-β-d-arabino-hexopyranoside (**7**)

To a solution of **6** (940 mg, 1.77 mmol) in dry DMF (19 mL) a solution of lithium azide (20% in water, 2.17 mL, 8.86 mmol) was added at 25 °C, and the solution was stirred at this temperature for 4 days. The solvents were removed under diminished pressure, and the remaining residue was dissolved in DCM (100 mL) and water (50 mL). The aq. phase was extracted with DCM (3 × 100 mL), and the combined organic layers were dried (Na_2_SO_4_). The solvent was removed under reduced pressure, and the remaining residue was subjected to chromatography (silica gel, hexane/ethyl acetate, 85:15) to afford **7** (700 mg, 89%) as a colorless oil; [*α*]_*D*_ = −33.01° (*c* 0.35, CHCl_3_); *R*_*ƒ*_ (*n*-hexane/ethyl acetate, 85:15) = 0.39; analysis calcd for C_23_H_25_O_4_F_2_N_3_ (445.44): C 62.01, H 5.66, N 9.43; found: C 61.80, H 5.81, N 9.25.

#### *n*-Propyl 6-amino-2,6-dideoxy-2,2-difluoro-β-d-glucopyranoside (**8**)

A solution of **7** (280 mg, 0.69 mmol) in dry MeOH (20.0 mL) containing palladium on charcoal (10%, 270 mg) was hydrogenated (30 °C, 2.43 atm, 48 h). The solution was filtered through a pad of Celite; the pad was rinsed with methanol (4 × 50 mL). The combined organic layers were dried (MgSO_4_), the solvent was removed under reduced pressure, and the residue was subjected to chromatography (silica gel, methanol/ethyl acetate, 20:80) to afford **8** (80 mg, 48%) as a white foam; [*α*]_*D*_ = −20.26° (*c* 1.19, MeOH); *R*_*ƒ*_ (methanol/ethyl acetate, 10:90) = 0.14; analysis calcd for C_9_H_17_O_4_F_2_N (241.23): C 44.81, H 7.10, N 5.81; found: C 44.69, H 7.32, N 5.69.

#### Allyl 2-O-benzyl-(R)-4,6-O-benzylidene-β-d-ribo-hexopyranoside-3-ulose (**10**)

To a mixture of dry DMSO (8.41 g, 107.64 mmol) and dry DCM (72.0 ml) at −78 °C, a solution of trifluoroacetic anhydride (16.05 g, 76.41 mmol) in dry DCM (18.0 mL) was slowly added, and the mixture was stirred at this temperature for 45 min. Then a solution of **9** (14.67 g, 36.82 mmol) in dry dichloromethane (40.0 mL) was added dropwise, maintaining the temperature at −78 °C during this addition. The mixture was stirred for 2 h at −78 °C, then a solution of Et_3_N (9.0 mL, 64.57 mmol) in dry DCM (40.0 ml) was added. The mixture was stirred at −78 °C for another 30 min and at 25 °C for 12 h. Usual aqueous workup followed by chromatography (silica gel, *n*-hexane/ethyl acetate, 85:15) gave **10** (10.90 g, 75%) as a white solid; MP 125–127 °C; [*α*]_*D*_ = −64.84° (*c* 0.49, CHCl_3_); *R*_*ƒ*_ (*n*-hexane/ethyl acetate, 5:3) = 0.73; analysis calcd for C_23_H_24_O_6_ (396.43): C 69.68, H 6.10; found: C 69.50, H 6.37.

#### Allyl 2-O-benzyl-(R)-4,6-O-benzylidene-3-deoxy-3,3-difluoro-β-d-ribo-hexopyranoside (**11**)

To a solution of **10** (300 mg, 0.72 mmol) in dry DCM (6.0 mL) DAST (417 μL, 3.03 mmol) was added dropwise, and the mixture was stirred at 25 °C for 5 days. Methanol (2.0 mL) was carefully added, and the solvents were removed under diminished pressure. Usual aqueous workup followed by chromatography (silica gel, *n*-hexane/ethyl acetate, 5:3) gave **11** (167 mg, 55%) as a white amorphous solid; [*α*]_*D*_ = −27.89° (*c* 0.39, CHCl_3_); *R*_*ƒ*_ (*n*-hexane/ethyl acetate, 5:3) = 0.67; analysis calcd for C_23_H_24_O_5_F_2_ (418.43): C 66.02, H 5.78; H 65.79, H 5.93.

#### Allyl 2,6-di-O-benzyl-3-deoxy-3,3-difluoro-β-d-ribo-hexopyranoside (**12**) and allyl 2,4-di-O-benzyl-3-deoxy-3,3-difluoro-β-d-ribo-hexopyranoside (**13**)

To an ice-cold solution of **11** (4.21 g, 10.06 mmol) in dry ether (40 mL) and THF (40 mL) lithium aluminum hydride (687 mg, 18.111 mmol) was added in several portions, and the suspension was stirred for 15 min at 25 °C. Then, the suspension was heated under reflux, and a solution of dry aluminum chloride (2.28 g, 17.11 mmol) in dry ether (30 mL) was added followed by stirring under reflux for 72 h. The suspension was cooled to 25 °C, methanol (30 mL) was carefully added, and stirring was continued for another 45 min. Usual aqueous workup followed by chromatography (silica gel, *n*-hexane/ethyl acetate, 5:3) gave **12** (490 mg, 12%) and **13** (1.82 g, 43%) each as a viscous oil;

Data for **12**: [*α*]_*D*_ = +14.57° (*c* 0.36, CHCl_3_); *R*_*ƒ*_ (*n*-hexane/ethyl acetate, 5:3) = 0.47; analysis calcd for C_23_H_26_O_5_F_2_ (420.45): C 65.70, H 6.23; found: C 65.59, H 6.46.

Data for **13**: [*α*]_*D*_ = −17.26° (*c* 0.42, CHCl_3_); *R*_*ƒ*_ (*n*-hexane/ethyl acetate, 5:3) = 0.61; analysis calcd for C_23_H_26_O_5_F_2_ (420.45): C 65.70, H 6.23; found: C 65.56, H 6.41.

#### Allyl 2,4-di-O-benzyl-3, 6-dideoxy-3,3-difluoro-6-iodo-β-d-ribo-hexopyranoside (**14**)

To a solution of **12** (1.82 g, 4.33 mmol) in toluene (40 mL) containing triphenylphosphane (2.50 g, 9.52 mmol) and imidazole (1.33 g, 19.48 mmol) iodine (2.20 g, 3.66 mmol) was added in several portions. After stirring at 90 °C for 1 h, the reaction mixture was decanted and the remaining oil was washed with ether (4 × 100 mL). The combined organic layers were evaporated, and the remaining residue was subjected to chromatography (silica gel, *n*-hexane/ethyl acetate, 85:15) to afford **14** (1.80 g, 78%) as a colorless oil; [*α*]_*D*_ = +23.79° (*c* 0.36, CHCl_3_); *R*_*ƒ*_ (*n*-hexane/ethyl acetate, 85:15) = 0.51; analysis calcd for C_23_H_25_O_4_F_2_I (530.34): C 52.09, H 4.75; found: C 51.84, H 4.93.

#### Allyl 6-azido-2,4-di-O-benzyl-3,6-dideoxy-3,3-difluoro-β-d-ribo-hexopyranoside (**15**)

To a solution of **14** (1.45 g, 2.73 mmol) in dry DMF (29 mL) a solution of lithium azide (20% in water; 3.35 mL, 13.67 mmol) was added at 25 °C, and the solution was stirred at this temperature for 4 days. The solvents were removed under diminished pressure, and the remaining residue was dissolved in DCM (100 mL) and water (50 mL). The aq. phase was extracted with DCM (3 × 100 ml), and the combined organic layers were dried (Na_2_SO_4_). The solvent was removed under reduced pressure, and the remaining residue was subjected to chromatography (silica gel, *n*-hexane/ethyl acetate, 85:15) to afford **15** (1.16 mg, 77%) as a colorless oil; [*α*]_*D*_ = +33.95° (*c* 0.40, CHCl_3_); *R*_*ƒ*_ (*n*-hexane/ethyl acetate, 5:3) = 0.48; analysis calcd for C_23_H_25_O_4_F_2_N_3_ (445.44): C 62.01, H 5.66, N 9.43; found: C 61.86, H 5.71, N 9.21.

#### *n*-Propyl 6-amino-3,6-dideoxy-3,3-difluoro-β-d-ribo-hexopyranoside (**16**)

A solution of **15** (200 mg, 0.45 mmol) in dry methanol (20.0 mL) containing palladium on charcoal (10%, 200 mg) was hydrogenated (25 °C, 2.43 atm, 24 h). Workup as described above followed by chromatography (silica gel, methanol/ethyl acetate, 20:80) gave **16** (100 mg, 93%) as a white foam; [*α*]_*D*_ = −27.95° (*c* 0.33, MeOH); *R*_*ƒ*_ (methanol/ethyl acetate, 20:80) = 0.13; analysis calcd for C_9_H_17_O_4_F_2_N (241.23): C 44.81, H 7.10, N 5.81; found: C 44.65, H 7.34, N 5.68.

## Supplementary information

Supplementary Materials

## Data Availability

The data sets generated during and/or analyzed during the current study are available from the corresponding author on reasonable request.
