# Supplementary material for: n-Propyl 6-amino-2,6-dideoxy-2,2-difluoro-β-d-glucopyranoside is a good inhibitor for the β-galactosidase from E. coli
Source: Med Chem Res. 2021 Mar 5;30(5):1099–107. doi: 10.1007/s00044-021-02715-8 (PMC7934981; doi:10.1007/s00044-021-02715-8)
Supplement: Supplementary file 1 — Supplementary Materials [file 44_2021_2715_MOESM1_ESM.pdf]

Supplementary Item

Click here to access/download;Supplementary Item;Supplementary material_rev_2.docx

Supplementary material

***n*-Propyl 6-amino-2,6-dideoxy-2,2-difluoro-β-D-glucopyranoside is a good inhibitor for the β-galactosidase from *E. coli*.**

Immo Serbian [1], Erik Prell [2], Claudia Fischer [1], Hans-Peter Deigner [3], René Csuk [1,*]

[1] Martin–Luther–University Halle-Wittenberg, Organic Chemistry, Kurt–Mothes_Str. 2, D-06120 Halle (Saale), Germany,

[2] University Hospital Halle (Saale), Department of Radiation Medicine, Section of Nuclear Medicine, Ernst–Grube Str. 40, D-06120 Halle (Saale), Germany,

[3] Furtwangen University, Medical and Life Sciences Faculty, Jakob–Kienzle Str. 17, D-78054 Villingen-Schwenningen, Germany.

Experimental

NMR spectra were recorded using the Varian spectrometers Gemini 2000 or Unity 500 (δ given in ppm, *J* in Hz, internal Me_4Si or internal CCl_3F), IR spectra [film or KBr pellet; signal intensities: s (strong), m (medium), w (weak)] on a Perkin-Elmer FT-IR spectrometer Spectrum 1000, MS spectra were taken on a Intectra GmbH AMD 402 (electron impact, 70 eV) or on a Thermo Electron Finnigan LCQ (electrospray, voltage 4.5 kV, sheath gas nitrogen) instrument.

Additional spectroscopic data

*Allyl 3-O-benzyl-(R)-4,6-O-benzylidene-β-D-glucopyranoside (**1**)*

IR (KBr): ν = 3364*s*, 3067*m*, 3034*m*, 2971*m*, 2931*m*, 2907*m*, 2860*s*, 1966*w*, 1735*s*, 1643*m*, 1586*m*, 1498*m*, 1452*s*, 1400*m*, 1367*s*, 1303*m*, 1283*m*, 1264*m*, 1209*m*, 1173*s*, 1093*s*, 1066*s*, 1030*s*, 1008*s*, 964*s*, 932*s*, 876*m*, 753*s*, 697*s*, 665*m*, 629*m*, 593*m*, 450*m* cm^{-1}; ^{1}H NMR (400 MHz, $CDCl_3$): δ = 7.48–7.46 (*m*, 2 H, Ph), 7.39–7.26 (*m*, 8 H, Ph), 5.93 (*dddd*, 1 H, $^{3}J_{H-2', H-1''}$ = 6.4, $^{3}J_{H-2', H-1'}$ = 5.3, $^{3}J_{H-2', H-3'}$ = 10.5, $^{3}J_{H-2', H-3''}$ = 17.2, H-2'), 5.56 (*s*, 1 H, C*H*-benzylidene), 5.32 (*dddd*, 1 H, $^{2}J_{H-3'', H-3'}$ = 1.4, $^{3}J_{H-3'', H-2'}$ = 17.2, H-3''), 5.22 (*dddd*, 1 H, $^{2}J_{H-3', H-3''}$ = 1.4, $^{3}J_{H-3', H-2'}$ = 10.5, H-3'), 4.95 (*d*, 1 H, $^{2}J_{H'', H'}$ = 11.7, CH''_2(OBn)), 4.79 (*d*, 1 H, $^{2}J_{H'', H'}$ = 11.7, CH''_2(OBn)), 4.44 (*d*, 1 H, $^{3}J_{1, 2}$ = 7.6, H-1), 4.36 (*dddd*, 1 H, $^{3}J_{H-1'', H-2'}$ = 5.3, $^{2}J_{H-1'', H-1'}$ = 12.8, H-1''), 4.33 (*dd*, 1 H, $^{3}J_{6B, 5}$ = 4.9, $^{2}J_{6B, 6A}$ = 10.5, H-6_B), 4.14 (*dddd*, 1 H, $^{3}J_{H-1', H-2'}$ = 6.4, $^{2}J_{H-1', H-1''}$ = 12.7, H-1'), 3.79 (*dd*, 1 H, $^{3}J_{6A, 5}$ = 10.1, $^{2}J_{6A, 6B}$ = 10.5, H-6_A), 3.67 (*m*, 2 H, H-3, H-4),

3.58 (*dd*, 1 H, $^3J_{2,1}$ = 7.6, $^3J_{2,3}$ = 9.5, H-2), 3.43 (*ddd*, 1 H, $^3J_{5,6B}$ = 4.9, $^3J_{5,4}$ = 9.1, $^3J_{5,6A}$ = 10.1, H-5) ppm; ^{13}C NMR (125 MHz, $CDCl_3$): δ = 138.3 (C_{ar}), 137.2 (Ph), 133.5 (C2'), 129.0 (C_{ar}), 128.4 (C_{ar}), 128.2 (C_{ar}), 128.0 (C_{ar}), 127.8 (C_{ar}), 126.0 (C_{ar}), 118.2 (C3'), 102.2 (C1), 101.3 (*C*H-benzylidene), 81.4 (C3), 80.2 (C4), 74.6 (*C*H_2(OBn)), 74.3 (C2), 70.5 (C1'), 68.7 (C6), 66.4 (C5) ppm; MS (ESI, MeOH): m/z (%) = 399.2 ($[M + H]^+$, 18), 416.1 ($[M + NH_4]^+$, 30), 421.3 ($[M + Na]^+$, 22), 433.9 ($[M_2 + K, HMeOH]^{2+}$, 3), 617.2 ($[M_3 + K, H]^{2+}$, 16), 818.9 ($[M_2 + Na]^+$, 100).

Allyl 3-O-benzyl-(R)-4,6-O-benzylidene-β-D-arabino-hexopyranoside-2-ulose (**2**)

IR (KBr): ν = 3440*s*, 3066*m*, 3035*m*, 2861*s*, 1750*s*, 1665*m*, 1576*s*, 1497*m*, 1452*s*, 1401*m*, 1365*s*, 1262*s*, 1187*s*, 1137*s*, 1094*s*, 1065*s*, 1030*s*, 1009*s*, 962*s*, 932*s*, 880*m*, 746*s*, 697*s*, 642*m*, 476*m*, 454*w* cm^{-1}; ^{1}H NMR (500 MHz, $CDCl_3$): δ = 7.50–7.28 (*m*, 10 H, Ph), 5.94 (*dddd*, 1 H, $^3J_{H-2', H-1''}$ = 5.9, $^3J_{H-2', H-1'}$ = 5.2, $^3J_{H-2', H-3'}$ = 10.3, $^3J_{H-2', H-3''}$ = 17.2, H-2'), 5.58 (*s*, 1 H, C*H*-benzylidene), 5.35 (*dddd*, 1 H, $^4J_{H-3'', H-1'}$ = 1.0, $^2J_{H-3'', H-3'}$ = 1.0, $^4J_{H-3'', H-1''}$ = 4.7, $^3J_{H-3'', H-2'}$ = 17.2, H-3''), 5.26 (*dddd*, 1 H, $^4J_{H-3', H-1'}$ = 0.6, $^4J_{H-3', H-1''}$ = 0.6, $^2J_{H-3', H-3''}$ = 4.7, $^3J_{H-3', H-2'}$ = 10.3, H-3'), 4.96 (*d*, 1 H, $^2J_{H'', H'}$ = 12.2, C*H*''$_2$(OBn)), 4.89 (*s*, 1 H, H-1), 4.75 (*d*, 1 H, $^2J_{H', H''}$ = 12.2, C*H*'$_2$(OBn)), 4.44 (*dd*, 1 H, $^3J_{6B,5}$ = 4.9, $^2J_{6B,6A}$ = 10.5, H-6$_B$), 4.39 (*dddd*, 1 H, $^4J_{H-1'', H-3''}$ = 0.6, $^4J_{H-1'', H-3'}$ = 1.0, $^3J_{H-1'', H-2'}$ = 5.2, $^2J_{H-1'', H-1'}$ = 12.9, H-1''), 4.24–4.18 (*m*, 2 H, H-4, H-1'), 3.96 (*dd*, 1 H, $^3J_{3,H}$ = 0.5, $^3J_{3,4}$ = 9.9, H-3), 3.85 (*dd*, 1 H, $^3J_{6A,5}$ = 10.2, $^2J_{6A,6B}$ = 10.5, H-6$_A$), 3.48–3.42 (*m*, 1 H, H-5) ppm; ^{13}C NMR (125 MHz, $CDCl_3$): δ = 196.5 (C=O (C-2)), 137.2 (Ph), 136.8 (C_{ar}), 132.9 (C-2'), 129.1 (C_{ar}), 128.5 (C_{ar}), 128.4 (C_{ar}), 128.3 (C_{ar}), 128.2 (C_{ar}), 128.2 (C_{ar}), 128.0 (C_{ar}), 127.9 (C_{ar}), 127.9 (C_{ar}), 126.0 (C_{ar}), 118.9 (C-3'), 101.1 (C-1), 99.5 (*C*H-benzylidene), 82.0 (C-3), 80.0 (C-4), 73.3 (*C*H_2(OBn)), 70.2 (C-1'), 68.6 (C-6), 66.5 (C-5) ppm; MS (ESI, MeOH): m/z (%) = 397.0 ($[M + H]^+$, 4), 414.1 ($[M + NH_4]^+$, 10), 419.3 ($[M + Na]^+$, 4), 446.1 ($[M + NH_4, MeOH]^+$, 28), 451.3 ($[M + Na, MeOH]^+$, 34), 815.0 ($[M_2 + Na]^+$, 40), 847.0 ($[M_2 + Na, MeOH]^+$, 94), 878.9 ($[M_2 + Na, (MeOH)_2]^+$, 100).

Allyl 3-O-benzyl-(R)-4,6-O-benzylidene-2-deoxy-2,2-difluoro-β-D-arabino-hexopyranoside (**3**)

IR (KBr): ν = 3442*m*, 3066*m*, 3033*m*, 2938*m*, 2886*m*, 2362*w*, 1734*m*, 1677*m*, 1651*mm*, 1497*m*, 1452*m*, 1436*m*, 1410*m*, 1386*m*, 1366*m*, 1336*m*, 1314 *m*, 1288*m*, 1243*s*, 1200*s*, 1084*s*, 1030*s*, 1000*s*, 970*s*, 939*m*, 867*m*, 778*m*, 743*s*, 722*m*, 696*s*, 651*m*, 561*m*, 520*m*, 488*m* cm^{-1}; ^{1}H NMR (400 MHz, $CDCl_3$): δ = 7.47–7.44 (*m*, 2 H, Ph), 7.41–7.40 (*m*, 6 H, Ph), 7.32–7.27 (*m*, 2 H, Ph), 5.91 (*dddd*, 1 H, $^3J_{H-2', H-1''}$ = 5.1, $^3J_{H-2', H-1'}$ = 6.5, $^3J_{H-2', H-3'}$ = 10.4, $^3J_{H-2', H-3''}$ = 17.2, H-2'), 5.56 (*s*, 1 H, C*H*-benzylidene), 5.35 (*dddd*, 1 H, $^4J_{H-3'', H-1'}$ = 1.5, $^2J_{H-3'', H-3'}$ = 1.5, $^4J_{H-3'', H-1''}$ = 1.5,

$^{3}J_{H\text{-}3'', H\text{-}2'}$ = 17.2, H-3''), 5.26 (*dddd*, 1 H, $^{4}J_{H\text{-}3', H\text{-}1'}$ = 1.2, $^{4}J_{H\text{-}3', H\text{-}1''}$ = 1.4, $^{2}J_{H\text{-}3', H\text{-}3''}$ = 1.5, $^{3}J_{H\text{-}3', H\text{-}2'}$ = 10.4, H-3'), 4.90 (*d*, 1 H, $^{2}J_{H'', H'}$ = 12.0, C*H*''$_2$(OBn)), 4.84 (*d*, 1 H, $^{2}J_{H', H''}$ = 12.0, C*H*'$_2$(OBn)), 4.57 (*d*, 1 H, $^{3}J_{1, F}$ = 14.9, H-1), 4.41 (*dddd*, 1 H, $^{4}J_{H\text{-}1'', H\text{-}3''}$ = 1.4, $^{4}J_{H\text{-}1'', H\text{-}3'}$ = 1.5, $^{3}J_{H\text{-}1'', H\text{-}2'}$ = 5.1, $^{2}J_{H\text{-}1'', H\text{-}1'}$ = 12.9, H-1''), 4.35 (*dd*, 1 H, $^{3}J_{6B, 5}$ = 5.0, $^{2}J_{6B, 6A}$ = 10.6, H-6$_B$), 4.19 (*dddd*, 1 H, $^{4}J_{H\text{-}1', H\text{-}3'}$ = 1.2, $^{4}J_{H\text{-}1', H\text{-}3''}$ = 1.5, $^{3}J_{H\text{-}1', H\text{-}2'}$ = 6.5, $^{2}J_{H\text{-}1', H\text{-}1''}$ = 12.9, H-1'), 3.91–3.76 (*m*, 3 H, H-3, H-6$_A$, H-4), 3.44 (*ddd*, 1 H, $^{3}J_{5, 6B}$ = 5.0, $^{3}J_{5, 4}$ = 9.7, $^{3}J_{5, 6A}$ = 9.6, H-5) ppm; ^{13}C NMR (125 MHz, CDCl$_3$): δ = 137.2 (C$_{ar}$), 136.9 (Ph), 132.7 (C-2'), 129.1 (C$_{ar}$), 128.3 (C$_{ar}$), 128.2 (C$_{ar}$), 128.1 (C$_{ar}$), 128.0 (C$_{ar}$), 126.0 (C$_{ar}$), 118.8 (C-3'), 116.1 (*dd*, $^{1}J_{2, F}$ = 254.6, $^{1}J_{2, F}$ = 258.7, C-2), 101.4 (*C*H-benzylidene), 97.8 (*dd*, $^{2}J_{1, F}$ = 19.9, $^{2}J_{1, F}$ = 28.3, C-1), 79.2 (*dd*, $^{2}J_{3, F}$ = 8.8, $^{2}J_{3, F}$ = 8.8, C-3), 77.5 (*dd*, $^{3}J_{4, F}$ = 18.1, $^{3}J_{4, F}$ = 258.2, C-4), 74.7 (*d*, $^{5}J_{CH2(OBn), F}$ = 1.1, *C*H$_2$(OBn)), 71.0 (C-1'), 68.3 (C-6), 66.5 (C5) ppm; ^{19}F NMR (188 MHz, CDCl$_3$): δ = -118.53 (*dd*, 1 F, $^{3}J_{F'', 3}$ = 5.2, $^{2}J_{F'', F'}$ = 252.2, F''), -136.37 (*ddd*, 1 F, $^{3}J_{F', 1}$ = 14.9, $^{3}J_{F', 3}$ = 20.1, $^{2}J_{F'', F'}$ = 252.2, F') ppm; MS (ESI, MeOH): m / z (%) = 419.2 ([M + H]$^+$, 28), 436.1 ([M + NH$_4$]$^+$, 57), 441.3 ([M + Na]$^+$, 56), 541.3 ([M + H, MeOH]$^+$, 18), 646.9 ([M$_3$ + K, H]$^{2+}$, 18), 858.7 ([M$_2$ + Na]$^+$, 100), 868.8 ([M$_2$ + H, MeOH]$^+$, 28).

*Allyl 3,6-di-O-benzyl-2-deoxy-2,2-difluoro-β-D-arabino-hexopyranoside (**4**)*

IR (film): ν = 3450*m*, 6064*m*, 3032*m*, 2876*m*, 1958*w*, 1723*w*, 1648*w*, 1605*w*, 1497*m*, 1455*m*, 1408*m*, 1363*m*, 1315*m*, 1260*m*, 1208*m*, 1183*m*, 1075*s*, 934*m*, 863*m*, 781*m*, 740*m*, 699*s*, 608*w* cm^{-1}; ^{1}H NMR (500 MHz, CDCl$_3$): δ = 7.40–7.27 (*m*, 10 H, Ph), 5.93 (*dddd*, 1 H, $^{3}J_{H\text{-}2', H\text{-}1''}$ = 5.1, $^{3}J_{H\text{-}2', H\text{-}1'}$ = 6.5, $^{3}J_{H\text{-}2', H\text{-}3'}$ = 10.5, $^{3}J_{H\text{-}2', H\text{-}3''}$ = 17.0, H-2'), 5.34 (*dddd*, 1 H, $^{4}J_{H\text{-}3'', H\text{-}1'}$ = 1.6, $^{2}J_{H\text{-}3'', H\text{-}3'}$ = 1.6, $^{4}J_{H\text{-}3'', H\text{-}1''}$ = 1.6, $^{3}J_{H\text{-}3'', H\text{-}2'}$ = 17.0, H-3''), 5.25 (*dddd*, 1 H, $^{4}J_{H\text{-}3', H\text{-}1'}$ = 1.3, $^{4}J_{H\text{-}3', H\text{-}1''}$ = 1.6, $^{2}J_{H\text{-}3', H\text{-}3''}$ = 1.6, $^{3}J_{H\text{-}3', H\text{-}2'}$ = 10.5, H-3'), 4.99 (*d*, 1 H, $^{2}J_{H'', H'}$ = 11.5, C*H*''$_2$(OBn)), 4.72 (*d*, 1 H, $^{2}J_{H', H''}$ = 11.5, C*H*'$_2$(OBn)), 4.61 (*d*, 1 H, $^{2}J_{H'', H'}$ = 12.0, C*H*''$_2$(OBn)), 4.56 (*d*, 1 H, $^{2}J_{H', H''}$ = 12.0, C*H*'$_2$(OBn)), 4.52 (*d*, 1 H, $^{3}J_{1, F'}$ = 14.8, H-1), 4.42 (*dddd*, 1 H, $^{4}J_{H\text{-}1'', H\text{-}3''}$ = 1.6, $^{4}J_{H\text{-}1'', H\text{-}3'}$ = 1.3, $^{3}J_{H\text{-}1'', H\text{-}2'}$ = 5.1, $^{2}J_{H\text{-}1'', H\text{-}1'}$ = 12.9, H-1''), 4.19 (*dddd*, 1 H, $^{4}J_{H\text{-}1', H\text{-}3'}$ = 1.6, $^{4}J_{H\text{-}1', H\text{-}3''}$ = 1.6, $^{3}J_{H\text{-}1', H\text{-}2'}$ = 6.5, $^{2}J_{H\text{-}1', H\text{-}1''}$ = 12.9, H-1'), 3.80 (*dd*, 1 H, $^{3}J_{6B, 5}$ = 3.8, $^{2}J_{6B, 6A}$ = 10.6, H-6$_B$), 3.77 (*ddd*, 1 H, $^{3}J_{5, 6B}$ = 3.8, $^{3}J_{5, 4}$ = 5.5, $^{3}J_{5, 6A}$ = 5.3, H-5), 3.74 (*dd*, 1 H, $^{3}J_{6A, 5}$ = 5.5, $^{2}J_{6A, 6B}$ = 10.6, H-6$_A$), 3.56 (*ddd*, 1 H, $^{3}J_{3, F''}$ = 5.2, $^{3}J_{3, 4}$ = 9.0, $^{3}J_{3, F'}$ = 19.1, H-3), 3.51 (*ddd*, 1 H, $^{3}J_{4, 3}$ = 9.0, $^{3}J_{4, 5}$ = 5.3, $^{4}J_{4, F'}$ = 4.3, H-4) ppm; ^{13}C NMR (125 MHz, CDCl$_3$): δ = 137.7 (Ph), 137.2 (Ph), 133.0 (C-2'), 129.0 (C$_{ar}$), 128.6 (C$_{ar}$), 128.6 (C$_{ar}$), 128.4 (C$_{ar}$), 128.2 (C$_{ar}$), 128.1 (C$_{ar}$), 127.8 (C$_{ar}$), 127.7 (C$_{ar}$), 127.6 (C$_{ar}$), 127.0 (C$_{ar}$), 118.6 (C-3'), 116.1 (*dd*, $^{1}J_{2, F}$ = 253.1, $^{1}J_{2, F}$ = 258.2, C-2), 96.8 (*dd*, $^{2}J_{1, F}$ = 19.4, $^{2}J_{1, F}$ = 27.4, C-1), 80.6 (*dd*, $^{2}J_{3, F}$ = 18.5, $^{2}J_{3, F}$ = 18.2, C-3), 75.0 (*d*, $^{4}J_{CH2(OBn), F}$ = 2.9, (*C*H$_2$(OBn)), 74.4 (C-5), 73.7 (*C*H$_2$(OBn)), 70.6 (C-1'), 70.2 (*d*, $^{3}J_{4,}$

$_{F}$ = 8.0, C-4), 69.8 (C6) ppm; ^{19}F NMR (188 MHz, $CDCl_3$): δ = -119.14 (*dd*, 1 F, $^{3}J_{F'', 3}$ = 5.2, $^{2}J_{F'', F'}$ = 248.4, F''), -138.39 (*dddd*, 1 F, $^{4}J_{F', 4}$ = 4.3, $^{3}J_{F', 1}$ = 14.8, $^{3}J_{F', 3}$ = 19.1, $^{2}J_{F'', F'}$ = 248.4, F') ppm; MS (ESI, MeOH: m/z (%) = 438.1 ([M + NH_4]$^{+}$, 100), 443.2 ([M + Na]$^{+}$, 72).

*Allyl 3,4-di-O-benzyl-2-deoxy-2,2-difluoro-β-D-arabino-hexopyranoside (**5**)*

IR (film): ν = 3456*m*, 3065*m*, 3032*m*, 2926*s*, 2877*m*, 2360*w*, 1957*w*, 1728*m*, 1648*w*, 1605*w*, 1497*m*, 1455*s*, 1407*m*, 1358*s*, 1313*m*, 1261*s*, 1211*m*, 1180*m*, 1087*s*, 1029*s*, 932*m*, 861*m*, 780*m*, 739*s*, 699*s*, 668*m*, 609*m*, 465*m* cm^{-1}; ^{1}H NMR (500 MHz, $CDCl_3$): δ = 7.40–7.24 (*m*, 10 H, Ph), 5.92 (*dddd*, 1 H, $^{3}J_{H\text{-}2', H\text{-}1''}$ = 5.2, $^{3}J_{H\text{-}2', H\text{-}1'}$ = 6.3, $^{3}J_{H\text{-}2', H\text{-}3'}$ = 10.4, $^{3}J_{H\text{-}2', H\text{-}3''}$ = 17.2, H-2'), 5.35 (*dddd*, 1 H, $^{4}J_{H\text{-}3'', H\text{-}1'}$ = 1.3, $^{2}J_{H\text{-}3'', H\text{-}3'}$ = 1.6, $^{4}J_{H\text{-}3'', H\text{-}1''}$ = 1.6, $^{3}J_{H\text{-}3'', H\text{-}2'}$ = 17.2, H-3''), 5.29 (*dddd*, 1 H, $^{4}J_{H\text{-}3', H\text{-}1'}$ = 1.3, $^{4}J_{H\text{-}3', H\text{-}1''}$ = 1.6, $^{2}J_{H\text{-}3', H\text{-}3''}$ = 1.6, $^{3}J_{H\text{-}3', H\text{-}2'}$ = 10.4, H-3'), 4.96 (*d*, 1 H, $^{2}J_{H'', H'}$ = 11.3, C*H''*$_2$(OBn)), 4.86 (*d*, 1 H, $^{2}J_{H'', H'}$ = 10.9, C*H''*$_2$(OBn)), 4.76 (*d*, 1 H, $^{2}J_{H', H''}$ = 11.3, C*H'*$_2$(OBn)), 4.62 (*d*, 1 H, $^{2}J_{H', H''}$ = 10.9, C*H'*$_2$(OBn)), 4.54 (*d*, 1 H, $^{3}J_{1, F'}$ = 14.7, H-1), 4.40 (*dddd*, 1 H, $^{4}J_{H\text{-}1'', H\text{-}3''}$ = 1.3, $^{4}J_{H\text{-}1'', H\text{-}3'}$ = 1.3, $^{3}J_{H\text{-}1'', H\text{-}2'}$ = 5.2, $^{2}J_{H\text{-}1'', H\text{-}1'}$ = 12.9, H-1''), 4.20 (*dddd*, 1 H, $^{4}J_{H\text{-}1', H\text{-}3'}$ = 1.6, $^{4}J_{H\text{-}1', H\text{-}3''}$ = 1.6, $^{3}J_{H\text{-}1', H\text{-}2'}$ = 6.3, $^{2}J_{H\text{-}1', H\text{-}1''}$ = 12.9, H-1'), 3.87 (*dd*, 1 H, $^{3}J_{6B, 5}$ = 2.6, $^{2}J_{6B, 6A}$ = 12.2, H-6$_B$), 3.78–3.69 (*m*, 3 H, H-3, H-5, H-6$_A$), 3.39 (*ddd*, 1 H, $^{3}J_{4, 3}$ = 9.0, $^{3}J_{4, 5}$ = 4.4, $^{4}J_{4, F'}$ = 2.6, H-4) ppm; ^{13}C NMR (125 MHz, $CDCl_3$): δ = 137.6 (Ph), 137.2 (Ph), 132.9 (C-2'), 128.4 (C_{ar}), 128.4 (C_{ar}), 128.3 (C_{ar}), 128.2 (C_{ar}), 128.2 (C_{ar}), 128.1 (C_{ar}), 128.0 (C_{ar}), 128.0 (C_{ar}), 118.7 (C-3'), 116.3 (*dd*, $^{1}J_{2, F}$ = 254.4, $^{1}J_{2, F}$ = 257.2, C-2), 97.0 (*dd*, $^{2}J_{1, F}$ = 19.1, $^{2}J_{1, F}$ = 27.8, C-1), 81.4 (*dd*, $^{2}J_{3, F}$ = 18.2, $^{2}J_{3, F}$ = 18.7, C-3), 75.8 (*d*, $^{3}J_{4, F}$ = 8.2, C-4), 75.4 (*C*H_2(OBn)), 75.3 (*d*, $^{4}J_{CH2(OBn), F}$ = 2.9, (*C*H_2(OBn)), 71.0 (C-5), 61.6 (C-1'), 69.8 (C6) ppm; ^{19}F NMR (188 MHz, $CDCl_3$): δ = -118.69 (*d*, 1 F, $^{2}J_{F'', F'}$ = 248.5, F''), -138.52 (*dddd*, 1 F, $^{4}J_{F', 4}$ = 2.6, $^{3}J_{F', 1}$ = 14.7, $^{3}J_{F', 3}$ = 18.8, $^{2}J_{F'', F'}$ = 248.5, F') ppm; MS (ESI, MeOH): m/z (%) = 420.9 ([M + H]$^{+}$, 4), 438.2 ([M + NH_4]$^{+}$, 100), 443.3 ([M + Na]$^{+}$, 62).

*Allyl 3,4-di-O-benzyl-2,6-dideoxy-2,2-difluoro-6-iodo-β-D-arabino-hexopyranoside (**6**)*

IR (film): ν = 3064*m*, 3031*m*, 2876*m*, 1727*m*, 1648*w*, 1605*w*, 1498*m*, 1455*m*, 1408*m*, 1359*m*, 1260*m*, 1182*m*, 1083*s*, 1028*m*, 935*m*, 861*m*, 778*m*, 739*m*, 699*m*, 464*m* cm^{-1}; ^{1}H NMR (500 MHz, $CDCl_3$): δ = 7.39–7.24 (*m*, 10 H, Ph), 5.95 (*dddd*, 1 H, $^{3}J_{H\text{-}2', H\text{-}1''}$ = 5.0, $^{3}J_{H\text{-}2', H\text{-}1'}$ = 6.8, $^{3}J_{H\text{-}2', H\text{-}3'}$ = 10.4, $^{3}J_{H\text{-}2', H\text{-}3''}$ = 17.2, H-2'), 5.40 (*dddd*, 1 H, $^{4}J_{H\text{-}3'', H\text{-}1'}$ = 1.7, $^{2}J_{H\text{-}3'', H\text{-}3'}$ = 1.6, $^{4}J_{H\text{-}3'', H\text{-}1''}$ = 1.6, $^{3}J_{H\text{-}3'', H\text{-}2'}$ = 17.3, H-3''), 5.29 (*dddd*, 1 H, $^{4}J_{H\text{-}3', H\text{-}1'}$ = 1.2, $^{4}J_{H\text{-}3', H\text{-}1''}$ = 1.6, $^{2}J_{H\text{-}3', H\text{-}3''}$ = 1.6, $^{3}J_{H\text{-}3', H\text{-}2'}$ = 10.4, H-3'), 4.97 (*d*, 1 H, $^{2}J_{H'', H'}$ = 11.2, C*H''*$_2$(OBn)), 4.90 (*d*, 1 H, $^{2}J_{H'', H'}$ = 11.0, C*H''*$_2$(OBn)), 4.74 (*d*, 1 H, $^{2}J_{H', H''}$ = 11.2, C*H'*$_2$(OBn)), 4.65 (*d*, 1 H, $^{2}J_{H', H''}$ = 11.0, C*H'*$_2$(OBn)), 4.56 (*d*, 1 H, $^{3}J_{1, F'}$ = 14.7, H-1), 4.46 (*dddd*, 1 H, $^{4}J_{H\text{-}1'', H\text{-}3''}$ = 1.7, $^{4}J_{H\text{-}1'', H\text{-}3'}$ = 1.2, $^{3}J_{H\text{-}1'', H\text{-}2'}$ =

5.0, $^{2}J_{H\text{-}1'', H\text{-}1'}$ = 12.9, H-1''), 4.25 (*dddd*, 1 H, $^{4}J_{H\text{-}1', H\text{-}3'}$ = 1.6, $^{4}J_{H\text{-}1', H\text{-}3''}$ = 1.6, $^{3}J_{H\text{-}1', H\text{-}2'}$ = 6.8, $^{2}J_{H\text{-}1', H\text{-}1''}$ = 12.9, H-1'), 3.76 (*ddd*, 1 H, $^{3}J_{3, F''}$ = 5.3, $^{3}J_{3, 4}$ = 9.0, $^{3}J_{3, F}$ = 19.2, H-3), 3.52 (*ddd*, 1 H, $^{3}J_{4, F'}$ = 1.3, $^{3}J_{4, 5}$ = 10.0, $^{3}J_{4, 3}$ = 9.0, H-4), 3.48 (*dd*, 1 H, $^{3}J_{6B, 5}$ = 2.4, $^{2}J_{6B, 6A}$ = 10.7, H-6$_{B}$), 3.29 (*ddd*, 1 H, $^{3}J_{5, 6B}$ = 2.4, $^{3}J_{5, 6A}$ = 8.0, $^{3}J_{5, 4}$ = 10.0, H-5), 3.17 (*dd*, 1 H, $^{3}J_{6A, 5}$ = 8.0, $^{2}J_{6A, 6B}$ = 10.7, H-6$_{A}$) ppm; ^{13}C NMR (100 MHz, $CDCl_3$): δ = 137.3 (Ph), 137.1 (Ph), 132.8 (C-2'), 128.5 (C_{ar}), 128.5 (C_{ar}), 128.3 (C_{ar}), 128.1 (C_{ar}), 128.1 (C_{ar}), 119.1 (C-3'), 116.4 (*dd*, $^{1}J_{2, F}$ = 254.0, $^{1}J_{2, F}$ = 257.1, C-2), 96.6 (*dd*, $^{2}J_{1, F}$ = 19.1, $^{2}J_{1, F}$ = 27.6, C-1), 81.2 (*dd*, $^{2}J_{1, F}$ = 18.5, $^{2}J_{1, F}$ = 18.4, C-3), 79.5 (*d*, $^{2}J_{4, F}$ = 8.4, C-4), 75.7 (*C*H_2(OBn)), 75.3 (*C*H_2(OBn)), 74.8 (C-5), 70.7 (C-1'), 4.5 (C6) ppm; ^{19}F NMR (188 MHz, $CDCl_3$): δ = -118.70 (*dd*, 1 F, $^{3}J_{F'', 3}$ = 5.3, $^{2}J_{F'', F'}$ = 248.5, F''), -138.52 (*dddd*, 1 F, $^{4}J_{F', 4}$ = 1.3, $^{3}J_{F', 1}$ = 14.7, $^{3}J_{F', 3}$ = 19.2, $^{2}J_{F'', F'}$ = 248.5, F') ppm; MS (ESI, MeOH): m/z (%) = 531.1 ($[M + H]^+$, 35), 553.3 ($[M + Na]^+$, 100).

Allyl 6-azido-3,4-di-O-benzyl-2,6-dideoxy-2,2-difluoro-β-D-arabino-hexopyranoside (7)

IR (film): ν = 3065*m*, 3032*m*, 2923*m*, 2876*m*, 2101*s*, 1733*w*, 1648*w*, 1606*w*, 1498*m*, 1455*m*, 1407*m*, 1358*m*, 1319*m*, 1262*s*, 1213*m*, 1181*m*, 1077*s*, 1029*s*, 996*s*, 931 *m*, 887*m*, 860*m*, 778*m*, 750*m*, 699*s*, 558*w*, 463*w* cm^{-1}; ^{1}H NMR (500 MHz, $CDCl_3$): δ = 7.39–7.21 (*m*, 10 H, Ph), 5.93 (*dddd*, 1 H, $^{3}J_{H\text{-}2', H\text{-}1''}$ = 5.1, $^{3}J_{H\text{-}2', H\text{-}1'}$ = 6.5, $^{3}J_{H\text{-}2', H\text{-}3'}$ = 10.5, $^{3}J_{H\text{-}2', H\text{-}3''}$ = 17.1, H-2'), 5.37 (*dddd*, 1 H, $^{4}J_{H\text{-}3'', H\text{-}1'}$ = 1.4, $^{2}J_{H\text{-}3'', H\text{-}3'}$ = 1.5, $^{4}J_{H\text{-}3'', H\text{-}1''}$ = 1.5, $^{3}J_{H\text{-}3'', H\text{-}2'}$ = 17.1, H-3''), 5.28 (*dddd*, 1 H, $^{4}J_{H\text{-}3', H\text{-}1'}$ = 1.2, $^{4}J_{H\text{-}3', H\text{-}1''}$ = 1.5, $^{2}J_{H\text{-}3', H\text{-}3''}$ = 1.5, $^{3}J_{H\text{-}3', H\text{-}2'}$ = 10.5, H-3'), 4.98 (*d*, 1 H, $^{2}J_{H'', H'}$ = 11.2, C*H*''$_2$(OBn)), 4.87 (*d*, 1 H, $^{2}J_{H'', H'}$ = 11.1, C*H*''$_2$(OBn)), 4.75 (*d*, 1 H, $^{2}J_{H', H''}$ = 11.2, C*H*'$_2$(OBn)), 4.57–4.54 (*m*, 2 H, C*H*'$_2$(OBn), H-1), 4.44 (*dddd*, 1 H, $^{4}J_{H\text{-}1'', H\text{-}3''}$ = 1.5, $^{4}J_{H\text{-}1'', H\text{-}3'}$ = 1.2, $^{3}J_{H\text{-}1'', H\text{-}2'}$ = 5.1, $^{2}J_{H\text{-}1'', H\text{-}1'}$ = 12.9, H-1''), 4.21 (*dddd*, 1 H, $^{4}J_{H\text{-}1', H\text{-}3'}$ = 1.5, $^{4}J_{H\text{-}1', H\text{-}3''}$ = 1.5, $^{3}J_{H\text{-}1', H\text{-}2'}$ = 6.5, $^{2}J_{H\text{-}1', H\text{-}1''}$ = 12.9, H-1'), 3.76 (*ddd*, 1 H, $^{3}J_{3, F''}$ = 5.2, $^{3}J_{3, 4}$ = 8.8, $^{3}J_{3, F'}$ = 19.4, H-3), 3.58 (*ddd*, 1 H, $^{3}J_{4, F'}$ = 1.2, $^{3}J_{4, 5}$ = 9.7, $^{3}J_{4, 3}$ = 8.8, H-4), 3.52 (*ddd*, 1 H, $^{3}J_{5, 6B}$ = 2.8, $^{3}J_{5, 6A}$ = 6.2, $^{3}J_{5, 4}$ = 9.7, H-5), 3.36–3.34 (*m*, 2 H, H-6$_{B}$, H-6$_{A}$) ppm; ^{13}C NMR (100 MHz, $CDCl_3$): δ = 137.2 (Ph), 137.1 (Ph), 132.7 (C-2'), 128.5 (C_{ar}), 128.4 (C_{ar}), 128.2 (C_{ar}), 128.1 (C_{ar}), 128.1 (C_{ar}), 118.8 (C-3'), 116.2 (*dd*, $^{1}J_{2, F}$ = 256.5, $^{1}J_{2, F}$ = 256.5, C-2), 96.7 (*dd*, $^{2}J_{1, F}$ = 20.9, $^{2}J_{1, F}$ = 27.4, C-1), 81.7 (*dd*, $^{2}J_{3, F}$ = 17.1, $^{2}J_{3, F}$ = 19.3, C-3), 77.3 (*m*, C-4), 75.4 (*C*H_2(OBn)), 75.3 (*C*H_2(OBn)), 75.0 (C-5), 70.5 (C-1'), 51.1 (C-6) ppm; ^{19}F NMR (188 MHz, $CDCl_3$): δ = -118.43 (*dd*, 1 F, $^{3}J_{F'', 3}$ = 5.2, $^{2}J_{F'', F'}$ = 249.5, F''), -138.52 (*dddd*, 1 F, $^{4}J_{F', 4}$ = 1.2, $^{3}J_{F', 1}$ = 14.6, $^{3}J_{F', 3}$ = 19.4, $^{2}J_{F'', F'}$ = 249.5, F') ppm; MS (ESI, MeOH): m/z (%) = 463.5 ($[M + NH_4]^+$, 68), 468.3 ($[M + Na]^+$, 100).

*n-Propyl 6-amino-2,6-dideoxy-2,2-difluoro-β-D-glucopyranoside (**8**)*

IR (film): ν = 3384*s*, 2361*w*, 1622*m*, 1384*s*, 1071*s*, 955*m*, 854*m*, 752*m*, 643*m* cm^{-1}; ^{1}H NMR (500 MHz, CD_3OD): δ = 4.69 (*d*, 1 H, $^{3}J_{1, F'}$ = 15.3, H-1), 3.92 (*m*, 1 H, H-1''), 3.69 (*ddd*, 1 H, $^{3}J_{3, F''}$ = 6.0, $^{3}J_{3, 4}$ = 9.2, $^{3}J_{3, F'}$ = 20.6, H-3), 3.62–3.51 (*m*, 2 H, H-1', H-5), 3.41 (*dd*, 1 H, $^{3}J_{6B, 5}$ = 3.1, $^{2}J_{6B, 6A}$ = 13.3, H-6_B), 3.36 (*m*, 1 H, H-4), 3.07 (*dd*, 1 H, $^{3}J_{6A , 5}$ = 8.8, $^{2}J_{6A , 6B}$ = 13.3, H-6_A), 1.68–1.61 (*m*, 2 H, H-2'', H-2'), 0.95 (*dd*, 3 H, $^{3}J_{H-3', H-2''/H-2'}$ = 7.4, $^{3}J_{H-3', H-2'\#/H-2'}$ = 7.4, H-3') ppm; ^{13}C NMR (125 MHz, CD_3OD): δ = 116.8 (*dd*, $^{1}J_{2, F}$ = 251.8, $^{1}J_{2, F}$ = 253.3, C-2), 99.6 (*dd*, $^{2}J_{1, F}$ = 19.2, $^{2}J_{1, F}$ = 27.8, C-1), 75.0 (*dd*, $^{2}J_{1, F}$ = 19.6, $^{2}J_{1, F}$ = 18.7, C-3), 73.5 (C-1'), 73.4 (C-5), 72.0 (*d*, $^{2}J_{4, F}$ = 7.7, C-4), 41.9 (C-6), 23.8 (C-2'), 10.6 (C-3') ppm; ^{19}F NMR (188 MHz, CD_3OD): δ = -124.03 (*dd*, 1 F, $^{3}J_{F'', 3}$ = 6.0, $^{2}J_{F'', F'}$ = 247.8, F''), -142.98 (*ddd*, 1 F, $^{3}J_{F', 1}$ = 15.3, $^{3}J_{F', 3}$ = 20.6, $^{2}J_{F'', F'}$ = 247.9, F') ppm; MS (ESI, MeOH): m/z (%) = 242.3 ($[M + H]^+$, 100).

Allyl 2-O-benzyl-(R)-4,6-O-benzylidene-β-D-glucopyranoside (**9**)

IR (KBr): ν = 3477*s*, 3288*s*, 3063*m*, 3030*m*, 2978*m*, 2877*s*, 1736*w*, 1645*m*, 1497*m*, 1454*s*, 1400*m*, 1384*s*, 1366*m*, 1348*m*, 1306*m*, 1266*m*, 1216*m*, 1180*s*, 1099*s*, 1046*s*, 1028*s*, 986*s*, 934*s*, 911*m*, 877*m*, 761*s*, 750*s*, 734*s*, 697*s*, 658*m*, 601*m*, 568*m*, 512*m*, 461*m* cm^{-1}; ^{1}H NMR (400 MHz, $CDCl_3$: δ = 7.47–7.45 (*m*, 2 H, Ph), 7.37–7.26 (*m*, 8 H, Ph), 5.93 (*dddd*, 1 H, $^{3}J_{H-2', H-1''}$ = 5.2, $^{3}J_{H-2', H-1'}$ = 5.7, $^{3}J_{H-2', H-3'}$ = 10.8, $^{3}J_{H-2', H-3''}$ = 17.3, H-2'), 5.50 (*s*, 1 H, C*H*-benzylidene), 5.34 (*dd*, 1 H, $^{2}J_{H-3'', H-3'}$ = 1.5, $^{3}J_{H-3'', H-2'}$ = 17.3, H-3''), 5.22 (*dd*, 1 H, $^{2}J_{H-3', H-3''}$ = 1.5, $^{3}J_{H-3', H-2'}$ = 10.8, H-3'), 4.94 (*d*, 1 H, $^{2}J_{H'', H'}$ = 12.0, CH''_2(OBn)), 4.72 (*d*, 1 H, $^{2}J_{H', H''}$ = 12.0, CH'_2(OBn)), 4.56 (*d*, 1 H, $^{3}J_{1, 2}$ = 7.7, H-1), 4.40 (*dd*, 1 H, $^{3}J_{H-1'', H-2'}$ = 5.2, $^{2}J_{H-1'', H-1'}$ = 12.7, H-1''), 4.32 (*dd*, 1 H, $^{3}J_{6B, 5}$ = 4.9, $^{2}J_{6B, 6A}$ = 10.5, H-6_B), 4.14 (*dd*, 1 H, $^{3}J_{H-1', H-2'}$ = 5.7, $^{2}J_{H-1', H-1''}$ = 12.7, H-1'), 3.82 (*dd*, 1 H, $^{3}J_{3, 2}$ = 9.1, $^{3}J_{3, 4}$ = 9.1, H-3), 3.76 (*dd*, 1 H, $^{3}J_{6A , 5}$ = 10.2, $^{2}J_{6A , 6B}$ = 10.5, H-6_A), 3.53 (*dd*, 1 H, $^{3}J_{4, 3}$ = 9.1, $^{3}J_{4, 5}$ = 9.6, H-4), 3.41 (*ddd*, 1 H, $^{3}J_{5, 6B}$ = 4.9, $^{3}J_{5, 4}$ = 9.6, $^{3}J_{5, 6A}$ = 10.2, H-5), 3.37 (*dd*, 1 H, $^{3}J_{2, 1}$ = 7.7, $^{3}J_{2, 3}$ = 9.1, H-2) ppm; ^{13}C NMR (125 MHz, $CDCl_3$): δ = 138.3 (C_{ar}), 137.0 (Ph), 133.6 (C-2'), 129.2 (C_{ar}), 128.5 (C_{ar}), 128.3 (C_{ar}), 128.2 (C_{ar}), 128.1 (C_{ar}), 128.0 (C_{ar}), 127.9 (C_{ar}), 126.3 (C_{ar}), 126.0 (C_{ar}), 117.7 (C-3'), 102.9 (C-1), 101.8 (*C*H-benzylidene), 81.8 (C-2), 80.4 (C-4), 74.8 (CH_2(OBn)), 73.2 (C-3), 70.7 (C-1'), 68.7 (C-6), 66.1 (C-5) ppm; MS (ESI, MeOH): *m*/*z* (%) = 399.1 ($[M H]^+$, 7), 416.1 ($[M+NH_4]^+$, 8), 421.3 ($[M+Na]^+$, 9), 617.2 ($[M_3+K, H]^{2+}$, 7), 818.9 ($[M_2+Na]^+$, 100).

*Allyl 2-O-benzyl-(R)-4,6-O-benzylidene-β-D-ribo-hexo-pyranoside-3-ulose (**10**)*

IR (KBr): ν = 3426*m*, 3066*m*, 3032*m*, 2882*m*, 1746*s*, 1645*m*, 1497*m*, 1452*m*, 1401*m*, 1384*m*, 1370*m*, 1362*m*, 1327*m*, 1276*m*, 1251*m*, 1216*m*, 1176*s*, 1162*m*, 1148*m*, 1128*s*, 1093*s*, 1074*s*,

1043*s*, 1030*s*, 1011*s*, 979*s*, 936*m*, 753*s*, 729*m*, 696*s*, 668*w*, 654*m*, 630*w*, 569*m* cm^{-1}; ^{1}H NMR (500 MHz, $CDCl_3$): δ = 7.49–7.47 (*m*, 2 H, Ph), 7.41–7.39 (*m*, 2 H, Ph), 7.36–7.26 (*m*, 6 H, Ph), 5.93 (*dddd*, 1 H, $^3J_{H-2', H-1''}$ = 5.3, $^3J_{H-2', H-1'}$ = 5.6, $^3J_{H-2', H-3'}$ = 10.8, $^3J_{H-2', H-3''}$ = 17.2, H-2'), 5.52 (*s*, 1 H, C*H*-benzylidene), 5.36 (*dddd*, 1 H, $^4J_{H-3'', H-1'}$ = 1.5, $^2J_{H-3'', H-3'}$ = 1.5, $^4J_{H-3'', H-1''}$ = 1.5, $^3J_{H-3'', H-2'}$ = 17.2, H-3''), 5.24 (*dddd*, 1 H, $^4J_{H-3', H-1'}$ = 1.4, $^4J_{H-3', H-1''}$ = 1.5, $^2J_{H-3', H-3''}$ = 1.5, $^3J_{H-3', H-2'}$ = 10.8, H-3'), 4.89 (*d*, 1 H, $^2J_{H'', H'}$ = 11.9, C*H''*$_2$(OBn)), 4.75 (*d*, 1 H, $^2J_{H', H''}$ = 11.9, C*H'*$_2$(OBn)), 4.72 (*d*, 1 H, $^3J_{1, 2}$ = 7.4, H-1), 4.45 (*dd*, 1 H, $^3J_{6B, 5}$ = 4.9, $^2J_{6B, 6A}$ = 10.4, H-6_B), 4.40 (*dddd*, 1 H, $^4J_{H-1'', H-3''}$ = 1.5, $^4J_{H-1'', H-3'}$ = 1.4, $^3J_{H-1'', H-2'}$ = 5.3, $^2J_{H-1'', H-1'}$ = 12.7, H-1''), 4.24–4.18 (*m*, 2 H, H-4, H-1'), 4.00 (*dd*, 1 H, $^3J_{2, 1}$ = 7.4, $^4J_{2, 4}$ = 1.5, H-2), 3.85 (*dd*, 1 H, $^3J_{6A, 5}$ = 9.9, $^2J_{6A, 6B}$ = 10.4, H-6_A), 3.57 (*ddd*, 1 H, $^3J_{5, 6B}$ = 4.9, $^3J_{5, 4}$ = 9.9, $^3J_{5, 6A}$ = 9.9, H-5) ppm; ^{13}C NMR (125 MHz, $CDCl_3$): δ = 196.4 (C=O (C-3)), 137.1 (C_{ar}), 136.3 (Ph), 133.2 (C-2'), 129.3 (C_{ar}), 128.8 (C_{ar}), 128.4 (C_{ar}), 128.3 (C_{ar}), 128.3 (C_{ar}), 128.4 (C_{ar}), 128.2 (C_{ar}), 128.1 (C_{ar}), 128.0 (C_{ar}), 127.9 (C_{ar}), 127.3 (C_{ar}), 126.3 (C_{ar}), 126.1 (C_{ar}), 118.1 (C-3'), 104.1 (C-1), 101.7 (*C*H-benzylidene), 82.8 (C-2), 81.8 (C-4), 73.6 (*C*H_2(OBn)), 71.0 (C-1'), 69.2 (C-6), 66.5 (C-5) ppm; MS (ESI–MeOH): m/z (%) = 397.0 ($[M + H]^+$, 6), 414.1 ($[M + NH_4]^+$, 14).

Allyl 2-O-benzyl-(R)-4,6-O-benzylidene-3-deoxy-3,3-difluoro-β-D-ribo-hexopyranoside ***(11)***
IR (film): ν = 2927*m*, 1736*m*, 1454*m*, 1384*m*, 1252*m*, 1093*s*, 745*w*, 698*m* cm^{-1}; ^{1}H NMR (500 MHz, $CDCl_3$): δ = 7.50–7.48 (*m*, 2 H, Ph), 7.41–7.28 (*m*, 8 H, Ph), 5.93 (*dddd*, 1 H, $^3J_{H-2', H-1''}$ = 0.7, $^3J_{H-2', H-1'}$ = 5.8, $^3J_{H-2', H-3'}$ = 10.5, $^3J_{H-2', H-3''}$ = 17.2, H-2'), 5.53 (*s*, 1 H, C*H*-benzylidene), 5.35 (*dddd*, 1 H, $^4J_{H-3'', H-1'}$ = 1.5, $^2J_{H-3'', H-3'}$ = 1.5, $^4J_{H-3'', H-1''}$ = 1.5, $^3J_{H-3'', H-2'}$ = 17.2, H-3''), 5.24 (*dddd*, 1 H, $^4J_{H-3', H-1'}$ = 1.3, $^4J_{H-3', H-1''}$ = 1.5, $^2J_{H-3', H-3''}$ = 1.3, $^3J_{H-3', H-2'}$ = 10.5, H-3'), 4.89 (*d*, 1 H, $^2J_{H'', H'}$ = 11.5, C*H''*$_2$(OBn)), 4.86 (*d*, 1 H, $^2J_{H', H''}$ = 11.5, C*H'*$_2$(OBn)), 4.70 (*dd*, 1 H, $^3J_{1, 2}$ = 7.8, $^4J_{1, F'}$ = 1.1, H-1), 4.42–4.37 (*m*, 2 H, H-6_B, H-1''), 4.18 (*dddd*, 1 H, $^4J_{H-1', H-3'}$ = 1.3, $^4J_{H-1', H-3''}$ = 1.5, $^3J_{H-1', H-2'}$ = 5.8, $^2J_{H-1', H-1''}$ = 12.7, H-1'), 3.79–3.68 (*m*, 3 H, H-6_A, H-4, H-5), 3.55 (*ddd*, 1 H, $^3J_{2, 1}$ = 7.8, $^3J_{2, F''}$ = 4.7, $^3J_{2, F}$ = 18.9, H-2) ppm; ^{13}C NMR (125 MHz, $CDCl_3$): δ = 137.1 (C_{ar}), 136.4 (Ph), 133.3 (C-2'), 129.3 (C_{ar}), 128.4 (C_{ar}), 128.3 (C_{ar}), 128.3 (C_{ar}), 128.0 (C_{ar}), 128.0 (C_{ar}), 127.8 (C_{ar}), 126.2 (C_{ar}), 117.9 (C-3'), 117.2 (dd, $^1J_{3, F}$ = 250.0, $^1J_{3, F}$ = 255.5, C-3), 101.9 (*C*H-benzylidene), 101.6 (*d*, $^3J_{1, F}$ = 5.7, C-1), 78.5 (*dd*, $^2J_{2, F}$ = 18.3, $^2J_{2, F}$ = 18.5, C-2), 77.9 (*dd*, $^2J_{4, F}$ = 18.5, $^2J_{4, F}$ = 18.9, C-4), 75.1 (*C*H_2(OBn)), 71.0 (C-1'), 68.7 (C6), 64.3 (*d*, $^3J_{5, F}$ = 6.9, C-5) ppm; ^{19}F NMR (188 MHz, $CDCl_3$): δ = -119.01 (*ddd*, 1 F, $^3J_{F'', 2}$ = 4.7, $^3J_{F'', 4}$ = 4.7, $^2J_{F'', F'}$ = 243.6, F''), -132.73 (*dddd*, 1 F, $^4J_{F', 1}$ = 1.1, $^3J_{F', 4}$ = 17.8, $^2J_{F', 2}$ = 18.9, $^2J_{F', F''}$ = 243.6, F') ppm; MS (ESI, MeOH): m/z (%) = 419.1 ($[M + H]^+$, 56), 436.1 ($[M + NH_4]^+$, 52), 441.3 ($[M + Na]^+$, 53), 647.1 ($[M_3 + K, H]^{2+}$, 18), 853.8 ($[M_2 + NH_4]^+$, 35), 858.7 ($[M_2 + Na]^+$, 100).

*Allyl 2,6-di-O-benzyl-3-deoxy-3,3-difluoro-β-*D*-ribo-hexopyranoside (**12**)*

IR (film): ν = 3444*m*, 3065*w*, 3032*w*, 2880*m*, 2360*w*, 1703*w*, 1648*w*, 1498*m*, 1455*m*, 1405*m*, 1353*m*, 1239*m*, 1175*m*, 1094*s*, 1038*s*, 930*m*, 861*w*, 739*m*, 699*m*, 668*w*, 610*w*, 544*w* cm^{-1}; ^{1}H NMR (500 MHz, $CDCl_3$): δ = 7.41–7.25 (*m*, 10 H, Ph), 5.92 (*dddd*, 1 H, $^{3}J_{\mathrm{H-2', H-1''}}$ = 5.4, $^{3}J_{\mathrm{H-2', H-1'}}$ = 5.8, $^{3}J_{\mathrm{H-2', H-3'}}$ = 10.5, $^{3}J_{\mathrm{H-2', H-3''}}$ = 17.2, H-2'), 5.33 (*dddd*, 1 H, $^{4}J_{\mathrm{H-3'', H-1'}}$ = 1.2, $^{2}J_{\mathrm{H-3'', H-3'}}$ = 1.6, $^{4}J_{\mathrm{H-3'', H-1''}}$ = 1.3, $^{3}J_{\mathrm{H-3'', H-2'}}$ = 17.2, H-3''), 5.22 (*dddd*, 1 H, $^{4}J_{\mathrm{H-3', H-1'}}$ = 1.2, $^{4}J_{\mathrm{H-3', H-1''}}$ = 1.3, $^{2}J_{\mathrm{H-3', H-3''}}$ = 1.6, $^{3}J_{\mathrm{H-3', H-2'}}$ = 10.5, H-3'), 4.90 (*d*, 1 H, $^{2}J_{\mathrm{H'', H'}}$ = 11.1, C*H''*$_2$(OBn)), 4.89 (*d*, 1 H, $^{2}J_{\mathrm{H'', H'}}$ = 11.4, C*H''*$_2$(OBn)), 4.84 (*d*, 1 H, $^{2}J_{\mathrm{H', H''}}$ = 11.4, C*H'*$_2$(OBn)), 4.65 (*d*, 1 H, $^{2}J_{\mathrm{H', H''}}$ = 11.1, C*H'*$_2$(OBn)), 4.62 (*dd*, 1 H, $^{4}J_{\mathrm{1, F'}}$ = 1.3, $^{3}J_{\mathrm{1, 2}}$ = 7.9, H-1), 4.37 (*dddd*, 1 H, $^{4}J_{\mathrm{H-1'', H-3''}}$ = 1.3, $^{4}J_{\mathrm{H-1'', H-3'}}$ = 1.3, $^{3}J_{\mathrm{H-1'', H-2'}}$ = 5.4, $^{2}J_{\mathrm{H-1'', H-1'}}$ = 12.8, H-1''), 4.16 (*dddd*, 1 H, $^{4}J_{\mathrm{H-1', H-3'}}$ = 1.2, $^{4}J_{\mathrm{H-1', H-3''}}$ = 1.2, $^{3}J_{\mathrm{H-1', H-2'}}$ = 5.8, $^{2}J_{\mathrm{H-1', H-1''}}$ = 12.9, H-1'), 3.85 (*dd*, 1 H, $^{3}J_{\mathrm{6B, 5}}$ = 1.7, $^{2}J_{\mathrm{6B, 6A}}$ = 12.1, H-6$_{\mathrm{B}}$), 3.71 (*dd*, 1 H, $^{3}J_{\mathrm{6A, 5}}$ = 3.6, $^{2}J_{\mathrm{6A, 6B}}$ = 12.1, H-6$_{\mathrm{A}}$), 3.68 (*ddd*, 1 H, $^{3}J_{\mathrm{4, F''}}$ = 3.5, $^{3}J_{\mathrm{4, 5}}$ = 9.8, $^{3}J_{\mathrm{4, F'}}$ = 19.4, H-4), 3.52 (*ddd*, 1 H, $^{3}J_{\mathrm{5, 6B}}$ = 1.7, $^{3}J_{\mathrm{5, 4}}$ = 9.8, $^{3}J_{\mathrm{5, 6A}}$ = 3.6, H-5), 3.46 (*ddd*, 1 H, $^{3}J_{\mathrm{2, F''}}$ = 4.0, $^{3}J_{\mathrm{2, 1}}$ = 7.9, $^{3}J_{\mathrm{2, F'}}$ = 20.2, H-2) ppm; ^{13}C NMR (100 MHz, $CDCl_3$): δ = 137.3 (Ph), 137.0 (Ph), 133.5 (C-2'), 128.5 (C_{ar}), 128.3 (C_{ar}), 128.3 (C_{ar}), 128.2 (C_{ar}), 128.2 (C_{ar}), 127.9 (C_{ar}), 120.5 (*dd*, $^{1}J_{\mathrm{3, F}}$ = 248.0, $^{1}J_{\mathrm{3, F}}$ = 253.1, C-3), 117.6 (C-3'), 100.9 (*dd*, $^{2}J_{\mathrm{1, F}}$ = 1.0, $^{2}J_{\mathrm{1, F}}$ = 10.3, C-1), 78.2 (*dd*, $^{2}J_{\mathrm{2, F}}$ = 18.1, $^{2}J_{\mathrm{2, F}}$ = 18.8, C-2), 74.9 (*C*H_2(OBn)), 74.9 (*C*H_2(OBn)), 74.3 (*dd*, $^{3}J_{\mathrm{4, F}}$ = 18.5, $^{3}J_{\mathrm{4, F}}$ = 18.9, C-4), 73.3 (*d*, $^{3}J_{\mathrm{5, F}}$ = 7.7, C-5), 70.8 (C-6), 61.3 (C-1') ppm; ^{19}F NMR (188 MHz, $CDCl_3$): δ = -112.09 (*ddd*, 1 F, $^{3}J_{\mathrm{F'', 4}}$ = 3.5, $^{3}J_{\mathrm{F'', 2}}$ = 4.0, $^{2}J_{\mathrm{F'', F'}}$ = 246.5, F''), -130.66 (*dddd*, 1 F, $^{4}J_{\mathrm{F', 1}}$ = 1.3, $^{3}J_{\mathrm{F', 4}}$ = 19.4, $^{3}J_{\mathrm{F', 2}}$ = 20.2, $^{2}J_{\mathrm{F', F''}}$ = 246.5, F') ppm; MS (ESI–MeOH): *m* / *z* (%) = 438.2 ($[M + NH_4]^+$, 100), 443.2 ($[M + Na]^+$, 50).

*Allyl 2,4-di-O-benzyl-3-deoxy-3,3-difluoro-β-*D*-ribo-hexopyranoside (**13**)*

IR (film): ν = 3386*m*, 3064*m*, 3031*m*, 2876*m*, 1703*w*, 1606*w*, 1497*m*, 1454*m*, 1406*m*, 1364*m*, 1238*m*, 1209*m*, 1178*m*, 1072*s*, 914*m*, 853*m*, 738*m*, 698*s*, 596*m*, 463*m* cm^{-1}; ^{1}H NMR (500 MHz, $CDCl_3$): δ = 7.40–7.28 (*m*, 10 H, Ph), 5.93 (*dddd*, 1 H, $^{3}J_{\mathrm{H-2', H-1''}}$ = 5.5, $^{3}J_{\mathrm{H-2', H-1'}}$ = 5.5, $^{3}J_{\mathrm{H-2', H-3'}}$ = 10.7, $^{3}J_{\mathrm{H-2', H-3''}}$ = 17.3, H-2'), 5.33 (*dddd*, 1 H, $^{4}J_{\mathrm{H-3'', H-1'}}$ = 1.5, $^{2}J_{\mathrm{H-3'', H-3'}}$ = 1.5, $^{4}J_{\mathrm{H-3'', H-1''}}$ = 1.5, $^{3}J_{\mathrm{H-3'', H-2'}}$ = 17.3, H-3''), 5.21 (*dddd*, 1 H, $^{4}J_{\mathrm{H-3', H-1'}}$ = 1.4, $^{4}J_{\mathrm{H-3', H-1''}}$ = 1.5, $^{2}J_{\mathrm{H-3', H-3''}}$ = 1.3, $^{3}J_{\mathrm{H-3', H-2'}}$ = 10.7, H-3'), 4.88 (*d*, 1 H, $^{2}J_{\mathrm{H'', H'}}$ = 11.5, C*H''*$_2$(OBn)), 4.82 (*d*, 1 H, $^{2}J_{\mathrm{H', H''}}$ = 11.5, C*H'*$_2$(OBn)), 4.62 (*d*, 1 H, $^{2}J_{\mathrm{H'', H'}}$ = 12.1, C*H''*$_2$(OBn)), 4.59 (*dd*, 1 H, $^{4}J_{\mathrm{1, F'}}$ = 1.3, $^{3}J_{\mathrm{1, 2}}$ = 8.0, H-1), 4.57 (*d*, 1 H, $^{2}J_{\mathrm{H', H''}}$ = 12.1, C*H'*$_2$(OBn)), 4.39 (*dddd*, 1 H, $^{4}J_{\mathrm{H-1'', H-3''}}$ = 1.4, $^{4}J_{\mathrm{H-1'', H-3'}}$ = 1.5, $^{3}J_{\mathrm{H-1'', H-2'}}$ = 5.5, $^{2}J_{\mathrm{H-1'', H-1'}}$ = 12.9, H-1''), 4.15 (*dddd*, 1 H, $^{4}J_{\mathrm{H-1', H-3'}}$ = 1.3, $^{4}J_{\mathrm{H-1', H-3''}}$ = 1.5, $^{3}J_{\mathrm{H-1', H-2'}}$ = 5.5, $^{2}J_{\mathrm{H-1', H-1''}}$ = 12.9, H-1'), 3.81 (*ddd*, 1 H, $^{3}J_{\mathrm{4, F''}}$ = 3.9, $^{3}J_{\mathrm{4, 5}}$ = 9.9, $^{3}J_{\mathrm{4, F'}}$ = 19.4, H-4),

3.77–3.75 (*m*, 2 H, H-6$_B$, H-6$_A$), 3.57 (*m*, 1 H, H-5), 3.49 (*ddd*, 1 H, $^3J_{2, F''}$ = 4.2, $^3J_{2, 1}$ = 8.0, $^3J_{2, F'}$ = 20.4, H-2) ppm; ^{13}C NMR (100 MHz, $CDCl_3$): δ = 137.6 (Ph), 137.3 (Ph), 133.6 (C-2'), 128.4 (C_{ar}), 128.2 (C_{ar}), 127.9 (C_{ar}), 127.8 (C_{ar}), 127.7 (C_{ar}), 119.4 (*dd*, $^1J_{3, F}$ = 245.6, $^1J_{3, F}$ = 253.1, C-3), 117.5 (C-3'), 100.9 (*d*, $^2J_{1, F}$ = 10.4, C-1), 77.9 (*dd*, $^2J_{2, F}$ = 18.7, $^2J_{2, F}$ = 18.3, C-2), 74.9 (*C*H_2(OBn)), 73.7 (*C*H_2(OBn)), 72.9 (*d*, $^3J_{5, F}$ = 6.8, C-5), 70.6 (C1'), 69.8 (*dd*, $^3J_{4, F}$ = 20.0, $^3J_{4, F}$ = 20.0, C-4), 69.2 (C6) ppm; ^{19}F NMR (188 MHz, $CDCl_3$): δ = -116.30 (*ddd*, 1 F, $^3J_{F'', 4}$ = 3.9, $^3J_{F'', 2}$ = 4.2, $^2J_{F'', F'}$ = 243.8, F''), -133.84 (*dddd*, 1 F, $^4J_{F', 1}$ = 1.6, $^3J_{F', 4}$ = 19.4, $^3J_{F', 2}$ = 20.4, $^2J_{F', F''}$ = 243.8, F') ppm; MS (ESI, MeOH): m/z (%) = 438.1 ($[M + NH_4]^+$, 100), 443.2 ($[M + Na]^+$, 70).

*Allyl 2,4-di-O-benzyl-3, 6-dideoxy-3,3-difluoro-6-iodo-β-D-ribo-hexopyranoside (**14**)*

IR (film): ν = 3065*m*, 3032*m*, 2879*m*, 2101*w*, 1954*w*, 1876*w*, 1648*w*, 1606*w*, 1497*m*, 1455*s*, 1407*m*, 1347*s*, 1302*m*, 1285*m*, 1238*s*, 1212*m*, 1188*m*, 1088*s*, 1028*s*, 998*s*, 931*m*, 864*m*, 819*w*, 777*m*, 739*s*, 698*s*, 662*m*, 610*w*, 545*m*, 487*w* cm^{-1}; ^{1}H NMR (500 MHz, $CDCl_3$): δ = 7.41–7.29 (*m*, 10 H, Ph), 5.95 (*dddd*, 1 H, $^3J_{H-2', H-1''}$ = 5.7, $^3J_{H-2', H-1'}$ = 5.8, $^3J_{H-2', H-3'}$ = 10.9, $^3J_{H-2', H-3''}$ = 17.2, H-2'), 5.36 (*dddd*, 1 H, $^4J_{H-3'', H-1'}$ = 1.5, $^2J_{H-3'', H-3'}$ = 1.5, $^4J_{H-3'', H-1''}$ = 1.5, $^3J_{H-3'', H-2'}$ = 17.2, H-3''), 5.24 (*dddd*, 1 H, $^4J_{H-3', H-1'}$ = 1.5, $^4J_{H-3', H-1''}$ = 1.5, $^2J_{H-3', H-3''}$ = 1.5, $^3J_{H-3', H-2'}$ = 10.9, H-3'), 4.95 (*d*, 1 H, $^2J_{H'', H'}$ = 11.1, C*H*''$_2$(OBn)), 4.90 (*d*, 1 H, $^2J_{H'', H'}$ = 11.6, C*H*''$_2$(OBn)), 4.83 (*d*, 1 H, $^2J_{H', H''}$ = 11.6, C*H*'$_2$(OBn)), 4.66 (*d*, 1 H, $^2J_{H', H''}$ = 11.1, C*H*'$_2$(OBn)), 4.63 (*dd*, 1 H, $^4J_{1, F'}$ = 1.1, $^3J_{1, 2}$ = 7.9, H-1), 4.41 (*dddd*, 1 H, $^4J_{H-1'', H-3''}$ = 1.5, $^4J_{H-1'', H-3'}$ = 1.5, $^3J_{H-1'', H-2'}$ = 5.7, $^2J_{H-1'', H-1'}$ = 12.9, H-1''), 4.18 (*dddd*, 1 H, $^4J_{H-1', H-3'}$ = 1.5, $^4J_{H-1', H-3''}$ = 1.5, $^3J_{H-1', H-2'}$ = 5.8, $^2J_{H-1', H-1''}$ = 12.9, H-1'), 3.50 (*ddd*, 1 H, $^3J_{2, F''}$ = 4.7, $^3J_{2, 1}$ = 7.9, $^3J_{2, F'}$ = 20.2, H-2), 3.48 (*m*, 1 H, H-4), 3.45 (*dd*, 1 H, $^3J_{6B, 5}$ = 3.3, $^2J_{6B, 6A}$ = 10.7, H-6$_B$), 3.31 (*m*, 1 H, H-5), 3.21 (*dd*, 1 H, $^3J_{6A, 5}$ = 6.7, $^2J_{6A, 6B}$ = 10.7, H-6$_A$) ppm; ^{13}C NMR (125 MHz, $CDCl_3$): δ = 137.2 (Ph), 136.8 (Ph), 133.4 (C-2'), 129.0 (C_{ar}), 128.5 (C_{ar}), 128.4 (C_{ar}), 128.3 (C_{ar}), 128.1 (C_{ar}), 127.9 (C_{ar}), 120.2 (*dd*, $^1J_{3, F}$ = 249.6, $^1J_{3, F}$ = 249.6, C-3), 117.9 (C-3'), 100.4 (*dd*, $^2J_{1, F}$ = 10.7, C-1), 78.6 (*m*, C-2, C-4), 75.1 (*C*H_2(OBn)), 74.9 (*C*H_2(OBn)), 71.8 (*d*, $^3J_{5, F}$ = 7.7, C-5), 70.6 (C-1'), 5.6 (C6) ppm; ^{19}F NMR (188 MHz, $CDCl_3$): δ = -111.66 (*dd*, 1 F, $^3J_{F'', 2}$ = 4.7, $^2J_{F'', F'}$ = 247.3, F''), -129.65 (*dddd*, 1 F, $^4J_{F', 1}$ = 1.1, $^3J_{F', 4}$ = 19.6, $^3J_{F', 2}$ = 20.2, $^2J_{F', F''}$ = 247.3, F') ppm; MS (ESI, MeOH): m/z (%) = 531.2 ($[M + H]^+$, 100), 553.1 ($[M + Na]^+$, 67).

*Allyl 6-azido-2,4-di-O-benzyl-3,6-dideoxy-3,3-difluoro-β-D-ribo-hexopyranoside (**15**)*

IR (film): ν = 3089*m*, 3065*m*, 3032*m*, 2919*m*, 2879*m*, 2104*s*, 1877*w*, 1735*w*, 1648*w*, 1606*w*, 1498*m*, 1455*s*, 1405*m*, 1353*s*, 1287*s*, 1239*s*, 1176*m*, 1095*s*, 1028*s*, 1001*s*, 932*m*, 892*m*, 859*m*,

740*s*, 699*s*, 676*m*, 610*m*, 546*m*, 465*m* cm^{-1}; ^{1}H NMR (500 MHz, $CDCl_3$): δ = 7.41–7.31 (*m*, 10 H, Ph), 5.93 (*dddd*, 1 H, $^{3}J_{H-2', H-1''}$ = 5.3, $^{3}J_{H-2', H-1'}$ = 5.9, $^{3}J_{H-2', H-3'}$ = 10.8, $^{3}J_{H-2', H-3''}$ = 17.2, H-2'), 5.34 (*dddd*, 1 H, $^{4}J_{H-3'', H-1'}$ = 1.5, $^{2}J_{H-3'', H-3'}$ = 1.5, $^{4}J_{H-3'', H-1''}$ = 1.5, $^{3}J_{H-3'', H-2'}$ = 17.2, H-3''), 5.23 (*dddd*, 1 H, $^{4}J_{H-3', H-1'}$ = 1.2, $^{4}J_{H-3', H-1''}$ = 1.5, $^{2}J_{H-3', H-3''}$ = 1.5, $^{3}J_{H-3', H-2'}$ = 10.8, H-3'), 4.92 (*d*, 1 H, $^{2}J_{H'', H'}$ = 11.2, CH''_2(OBn)), 4.90 (*d*, 1 H, $^{2}J_{H'', H'}$ = 11.5, CH''_2(OBn)), 4.84 (*d*, 1 H, $^{2}J_{H', H''}$ = 11.5, CH'_2(OBn)), 4.62 (*dd*, 1 H, $^{4}J_{1, F'}$ = 1.2, $^{3}J_{1, 2}$ = 8.0, H-1), 4.59 (*d*, 1 H, $^{2}J_{H', H''}$ = 11.2, CH'_2(OBn)), 4.39 (*dddd*, 1 H, $^{4}J_{H-1'', H-3''}$ = 1.2, $^{4}J_{H-1'', H-3'}$ = 1.5, $^{3}J_{H-1'', H-2'}$ = 5.3, $^{2}J_{H-1'', H-1'}$ = 12.8, H-1''), 4.16 (*dddd*, 1 H, $^{4}J_{H-1', H-3'}$ = 1.5, $^{4}J_{H-1', H-3''}$ = 1.5, $^{3}J_{H-1', H-2'}$ = 5.9, $^{2}J_{H-1', H-1''}$ = 12.8, H-1'), 3.66 (*m*, 1 H, H-5), 3.58–3.44 (*m*, 3 H, H-4, H-2, H-6_B), 3.33 (*dd*, 1 H, $^{3}J_{6A, 5}$ = 5.9, $^{2}J_{6A, 6B}$ = 13.2, H-6_A) ppm; ^{13}C NMR (100 MHz, $CDCl_3$): δ = 137.3 (Ph), 136.7 (Ph), 133.4 (C-2'), 128.5 (C_{ar}), 128.3 (C_{ar}), 128.3 (C_{ar}), 128.1 (C_{ar}), 127.9 (C_{ar}), 122.2 (*dd*, $^{1}J_{3, F}$ = 248.6, $^{1}J_{3, F}$ = 252.9, C-3), 117.7 (C-3'), 100.6 (*dd*, $^{2}J_{1, F}$ = 10.4, C-1), 78.2 (*dd*, $^{2}J_{2, F}$ = 18.8, $^{2}J_{2, F}$ = 18.4, C-2), 75.3 (*dd*, $^{3}J_{4, F}$ = 19.1, $^{3}J_{4, F}$ = 18.8, C-4), 75.0 (CH_2(OBn)), 74.9 (*d*, $^{4}J_{CH2(OBn), F}$ = 3.5, CH_2(OBn)), 72.7 (*d*, $^{3}J_{5, F}$ = 8.0, C-5), 70.5 (C-1'), 51.1 (C-6) ppm; ^{19}F NMR (188 MHz, $CDCl_3$): δ = -111.98 (*d*, 1 F, $^{2}J_{F'', F'}$ = 247.5, F''), -130.66 (*dddd*, 1 F, $^{4}J_{F', 1}$ = 1.2, $^{3}J_{F', 4}$ = 19.8, $^{3}J_{F', 2}$ = 19.8, $^{2}J_{F', F''}$ = 247.5, F') ppm; MS (ESI, MeOH): m/z (%) = 463.5 ($[M + NH_4]^+$, 100), 468.3 ($[M + Na]^+$, 78).

*n-Propyl 6-amino-3,6-dideoxy-3,3-difluoro-β-*D*-ribo-hexopyranoside (**16**)*

IR (film): ν = 3458*w*, 2515*w*, 1639*w*, 1414*m*, 1319*s*, 1111*w*, 1063*m*, 1040*m*, 972*s*, 881*w*, 820*m*, 741*w*, 590*w*, 503*w*, 467*w*, 445*w* cm^{-1}; ^{1}H NMR (500 MHz, CD_3OD): δ = 4.46 (*dd*, 1 H, $^{4}J_{1, F'}$ = 1.7, $^{3}J_{1, 2}$ = 8.0, H-1), 3.91 (*ddd*, 1 H, $^{3}J_{H-1'', H-2'}$ = 3.1, $^{3}J_{H-1'', H-2''/H-2'}$ = 6.7, $^{3}J_{H-1'', H-3''}$ = 9.3, H-1''), 3.70–3.56 (*m*, 3 H, H-2, H-4, H-5), 3.53 (*ddd*, 1 H, $^{3}J_{H-1', H-2''/H-2}$ = 3.2, $^{3}J_{H-1', H-2''/H-2}$ = 6.6, $^{3}J_{H-1', H-3''}$ = 9.3, H-1'), 3.38 (*dd*, 1 H, $^{3}J_{6B, 5}$ = 3.1, $^{2}J_{6B, 6A}$ = 13.2, H-6_B), 3.09 (*dd*, 1 H, $^{3}J_{6A, 5}$ = 8.6, $^{2}J_{6A, 6B}$ = 13.2, H-6_A), 1.64 (*m*, 2 H, H-2'', H-2'), 0.95 (*m*, 3 H, H-3'); ^{13}C NMR (125 MHz, CD_3OD): δ = 119.0 (*m*, C-3), 101.5 (*d*, $^{3}J_{1, F}$ = 10.1, C-1), 71.4 (C-1'), 71.4–69.6 (*m*, C-2, C-4, C-5), 40.4 (C-6), 22.4 (C-2'), 9.3 (C-3') ppm; ^{19}F NMR (188 MHz, CD_3OD): δ = -119.49 (*d*, 1 F, $^{2}J_{F'', F'}$ = 244.8, F''), -138.00 (*ddd*, 1 F, $^{3}J_{F', 4}$ = 18.8, $^{3}J_{F', 2}$ = 18.8, $^{2}J_{F', F''}$ = 244.8, F') ppm; MS (ESI, MeOH): m/z (%) = 242.2 ($[M + H, 100]^+$).
